# Supplementary material for: Identification of gene function based on models capturing natural variability of Arabidopsis thaliana lipid metabolism
Source: Nat Commun. 2023 Aug 14;14:4897. doi: 10.1038/s41467-023-40644-9 (PMC10425450; doi:10.1038/s41467-023-40644-9)
Supplement: Supplementary file 1 — Supplementary Information [file 41467_2023_40644_MOESM1_ESM.pdf]

Identification of gene function based on models capturing natural variability of *Arabidopsis thaliana* lipid metabolism

Correa Córdoba *et al.*

Supplementary Method 1. Reconstruction of the lipid metabolic network of Arabidopsis leaves

The reconstructed lipid metabolic network, referred to as the "Plant Lipid Module" consists of 5956 reactions, 3108 metabolites and 16 compartments. For the model plant *Arabidopsis thaliana*, there are numerous data sources[1–10] and pathways databases[8,11–14] that facilitate the usage of automated tools from the constraint-based modeling framework to perform a top-down reconstruction of lipid metabolism. However, such a strategy would not be optimal given the evidence that annotation of lipid metabolism in plants is incomplete. For example, we reviewed the records that still require manual annotation in the UniProt Knowledgebase (UniProtKB) by searching specifically for genes associated with enzyme-catalyzed reactions related to lipid pathways. For 1995 of such genes in *A. thaliana*, 42% are assigned a score of 1 and 50% a score of 2, which correspond to the entries with rather basic annotation (e.g. "protein uncertain" and "protein predicted", respectively). For the case of the manually annotated 1106 genes, 20% and 24% are assigned a score of 2 and 3 (protein inferred by homology), respectively. We also compared the existing plant metabolic models of small[15–31] and medium size[32–38] as well as genome-scale models (GEMs)[39–55], and found that most of the metabolic reconstructions of small and medium size have low level of detail of the lipid network, since the lipid-related reactions are lumped. The exception is the metabolic reconstruction for rapeseed[33,34], with a total of 572 reactions of which 187 (32.7%) are lipid-related. After reviewing the published GEMs, we found that fatty acyl (FA) synthesis and lipid degradation reactions were detailed; however, the reactions of structural lipids synthesis (e.g. galactolipids and sulfolipids) and reactions of phospholipids (PLs) formation were lumped to a different extent. Further, we found that in most of the examined GEMs, the endoplasmic reticulum (ER) compartment was not included, although important reactions of glycerolipid formation, acyl editing and PLs synthesis, among others, take place in this compartment. We also identified that storage lipid formation (i.e., triacylglycerol (TG)) was represented by a single lumped reaction. Exceptions are the rice GEM (iOS2164)[53] and the evidence-based maize GEM[54], in which the ER compartment contained reactions of FA elongation, glycerolipid and glycerophospholipid synthesis, sphingolipid metabolism and sterol synthesis. For these reasons, we decided not to make use of these automated tools and to perform a bottom-up reconstruction.

Diverse bibliomic data were examined to allow up-to-date accurate reconstruction. The topology of the lipid biosynthesis network was refined with the aid of different resources such as the Aralipid website (http://aralip.plantbiology.msu.edu/)[12], the Kyoto Encyclopedia of Genes and Genomes (KEGG), the enzyme repository BRENDA, the universal protein database (UniProt), the Arabidopsis

Information Resource (TAIR) (https://www.arabidopsis.org/), the Arabidopsis Information Portal (https://www.araport.org)[56] and the central resource for Arabidopsis protein subcellular location data (SUBA) (https://suba.live/)[2].

A complete representation was made for reactions of FA synthesis and elongation in plastid and mitochondria, which includes the sequential condensation, reduction, dehydration, and reduction reactions taking place at each condensation cycle, in addition to the steps for FA desaturation and export from plastid. The model also includes the synthesis of octanoic acid in mitochondria, which is one of the major FAs in this compartment and is used in turn for the synthesis of lipoylated proteins (e.g. pyruvate dehydrogenase (PDH), α-ketoglutarate dehydrogenase (KGDH), and branched chain α-keto acid dehydrogenase (BCKADH) enzyme complexes.

The Plant Lipid Module also includes the prokaryotic and eukaryotic galactolipid, sulfolipid and PLs synthesis routes. Here, we note that although the TGD (1-5) proteins are part of the machinery involved in the transport of lipids from the ER to plastids, the identity of the lipid species transported which act as precursors for the eukaryotic pathway is still debated. Phosphatidic acid (PA) is considered as one candidate for transport, as TGD2 and TGD4 are able to bind PA[57,58]; in addition PA can support multiple membrane conformations and is a non-bilayer-forming lipid, requiring less energy for its removal from membranes[59,60]. However, PA binding to TGD proteins might also reflect its regulatory role exerted on MGD1, rather than a PA transport activity[61–63]. Several other molecules have been suggested as candidates for transport, including: diacylglycerol (DG), phosphatidylcholine (PC) and lyso-PC (LPC)[59,64,65]. DG like PA, it is a non-bilayer-forming lipid, and it also has the ability to flip between the leaflets of a bilayer at biologically relevant rates[59,66]. PC and lyso-PC have both been proposed as candidates for transport owing to the inability of chloroplasts to produce PC; however, this hypotheses would be relevant for TGD4 as PC is present only in the outer chloroplast envelope[59,67–69]. Due to the lack of consensus on the identity of the transported lipid moieties, we assume PC as the precursor. There is evidence that ALA10 is a P4-type ATPase that belongs to the family of flippases, which play a role in PC transfer from the ER to plastids when interacts with ALIS5 (ALA-interacting subunit 5)[61,65,70,71]. It has been proposed that ALA10 might create a specific lipid environment in the ER favoring lipid transfer by flipping specific PC species from the luminal to the cytosolic leaflet of the ER[71]. Once in the plastid envelope, it has been suggested that PC is first hydrolyzed by phospholipases (e.g. phospholipase D (PLDζ)) to form PA, which activates the MGDG synthase 1 (MGD1) and promotes galactoglycerolipids and plastid membranes biogenesis[61,71,72]. PA in turn is then transported to the inner envelope by binding the TDG proteins, to be later

dephosphorylated to DG by PA phosphatase (PP). Several enzymes with phosphatidate phosphatase activity have been identified to be located at the chloroplast inner membrane of *A. thaliana*[73].

The pathways in the ER include the *de novo* assembly of FA into the glycerol backbone (Kennedy pathway), which requires the participation of different enzymes, such as Acyl-CoA:sn-glycerol-3-phosphate acyltransferase (GPAT) that catalyzes the first acylation in position sn-1, to form lysophosphatidic acid (LPA), followed by the subsequent, acyl-CoA: lysophosphatidic acid acyltransferase (LPAAT), catalyzing the acylation at the sn-2 position of glycerol, to form PA. The phosphate in the sn-3 position of the PA is removed before the final step of acylation by the catalytic action of phosphatidic acid phosphatase (PAP), to form DG. Finally, acylation in the sn-3 position is catalyzed by acyl-CoA: diacylglycerol acyltransferase (DGAT), resulting in triacylglycerol (TG)[74–79]. The participation of glycerolipid species (e.g. DG) as a branch point to generate other lipid species (e.g. PLs)[78] is also represented in the Lipid Module, besides the more complex routes for the assembly of TG, in which PC is a central intermediate for the production of TG containing high levels of modified FA (e.g. polyunsaturated fatty acyls (PUFA))[80]. Hence, the Plant Lipid Module also incorporates the three known mechanisms that allow the flux of FA through PC for the eventual synthesis of TG: (1) enrichment of the acyl-CoA pool with modified FA resulting from acyl editing, which after their hydrolysis from PC by the action of phospholipase A2 (PLA2) are acylated into DG to form TG, (2) direct transfer of one FA from PC to DG to produce TG by the action of the phospholipid:1,2-diacyl-sn-glycerol-acyltransferase (PDAT) enzyme, and (3) the reverse transfer of 18:2 and 18:3 into TG catalyzed by the phosphatidylcholine:diacylglycerol cholinephosphotransferase (PDCT) enzyme, which transfers the phosphocholine headgroup from PC to DG that also constitutes a major reaction for the transfer of 18:1 into phosphatidylcholine for desaturation[80,81]. In respect to acyl editing, we would like to emphasize that PC is assumed as the major substrate for desaturation and acyl editing, since two lyso-PE acyltransferases (LPEAT) have also been identified, phosphatidylethanolamine (PE) has a minor role in the acyl editing that occurs during initial PLs assembly and LPEAT may instead be involved in membrane maintenance. As acyl editing involves a dynamic FA exchange predominantly between the sn-2 (but also sn-1) position of PC and acyl-CoA pools, the Plant Lipid Module includes the reverse reaction catalyzed by LPCAT and the FA hydrolysis from PC catalyzed by PLA2, followed by the activation of free-FA into CoA esters by LACS.

Other lipid-related pathways in the ER include the formation of sterols (e.g. episterol, avenasterol, campesterol, sitosterol, stigmasterol, brassicasterol) and the extra-plastidial FA elongation to form very-long-chain fatty acyl-CoAs (VLCFAs) up to 34 carbon atoms, the latter being used for cuticular

wax and sphingolipids synthesis. The wax biosynthetic pathways include the alkane-forming pathway (also known as the decarbonylation pathway) that produces aldehydes, alkanes, secondary alcohols and ketones, and a primary alcohol forming pathway (also known as the acyl reduction pathway) that produces primary alcohols and wax esters[12]. All the necessary reactions for the biosynthesis of sphingobases and ceramides are included, besides the representation of the formation of the two distinct pools of ceramide resulting from their synthesis in the ER. While one pool is glycosylated by glucosylceramide synthase (GCS), the other is transported to the Golgi apparatus where it receives a phosphorylinositol headgroup from phosphatidylinositol through the action of inositolphosphorylceramide synthase (IPCS) to form glycosyl inositol phosphorylceramide (GIPC) [12]. Since plant GIPCs can contain unique glycan decorations that include a conserved glucuronic acid (GlcA) residue and various additional sugars, we incorporated the reactions for the transfers of GlcA from UDP-GlcA to GIPCs by the action of glucuronosyltransferase and reactions for the addition of different sugar moieties (e.g. glucose, galactose, mannose, arabinose) catalyzed by glycosylinositolphosphorylceramide synthase[82–85].

The pathway for TG degradation was also fully included and comprised the removal of acyl chains from glycerol by the activity of lipases, followed by FAs transport to the peroxymes where the core β-oxidation cycle takes place to break down saturated and unsaturated FA molecules into acyl-CoA. The glyoxylate cycle is also part of the reactions added to the peroxisome, in addition to the synthesis of oxylipids as important precursors for jasmonates[86].

Although mitochondrial membranes contain PC, PE, phosphatidylinositol (PI) and cardiolipin (CL) as major PLs constituents, PC, PE and PI are mainly synthesized in the ER and transported to mitochondria, hence the Plant Lipid Module includes the biosynthetic routes for PC, PG, PI, phosphatidylserine (PS) and PE taking place in the ER, and the respective transport reactions between ER and mitochondria for the aforementioned PLs with exception of PE, wherein the latter is synthesized in the mitochondria from ER-imported PS by the action of PS decarboxylase. PLs transport among ER and mitochondria is assumed to occur via membrane contact sites (MCSs) since numerous MCSs have been observed between the mitochondria and the ER[65,87,88]. Since it has been suggested that CL is synthesized *de novo* in mitochondria from glycerol-3-phosphate and acyl-ACP, the necessary reactions leading to the synthesis of this lipid class were added to this compartment in addition to the FA assembly reaction into glycerol leading to the formation of CDP-DG and phosphatidylglycerol (PG) as CL precursors. Import of CDP-DG and PG from ER was also included since part of PG and CDP-DG synthesized in the ER might be used for CL biosynthesis.

Additional pathways were included for the formation of terpenoids in plastids. Moreover, we also included reactions representing the lipid remodeling events taking place under conditions such as cold, heat shock and phosphate starvation, which therefore allows performing simulations for these environments.

To simulate remodeling events under cold stress, the enzyme coded by the gene SENSITIVE TO FREEZING 2 (SFR2) was included, which encodes a galactolipid remodeling enzyme of the outer chloroplast envelope membrane that processively transfers galactosyl residues from the abundant monogalactolipid to different galactolipid acceptors, forming oligogalactolipids and diacylglycerol. In the Plant Lipid Module monogalactosyldiacylglycerol (MGDG) galactosyl residues are transferred to a second MGDG molecule to form digalactosyldiacylglycerol (DGDG) and DG. TG is concomitantly produced from the resultant DG and stored in the lipoprotein structures localized in the plastid known as plastoglobules [89,90]. The combined activity of SFR2 and TG-biosynthetic enzymes leads to the removal of monogalactolipids from the envelope membrane, changing the ratio of bilayer- to nonbilayer–forming membrane lipids. This SFR2-based mechanism compensates for changes in organelle volume and stabilizes membranes during freezing[91,92]. During the freezing and post-freezing recovery period hydrolysis of plastidic lipids has been reported; thus, reactions of MGDG hydrolysis were included[93].

Lipid remodeling under heat-shock stress was represented in the Plant Lipid Module by including the activity of a lipase designated HEAT INDUCIBLE LIPASE1 (HIL1) that induces the catabolism of MGDG, with particular preference towards MGDG containing polyunsaturated acyl groups, such as α-linolenate (18:3) and hexadecatrienoate (16:3) [94]. Since the expression of PES1[95] was reported to be involved in TG synthesis in chloroplasts, and was found to be increased under heat stress and during recovery[96]; hence, reactions for PUFAs removal from MGDG and their concomitant recycling into TG[94] were also included.

During plant growth on phosphate-limiting soils, the replacement of phospholipids by galacto- and sulfolipids in plant membranes represents an important adaptive process, where the non-specific phospholipase C5 (NPC5) is required for normal accumulation of DGDG during phosphate limitation in leaves[97]. The soluble NPC5 is reported to have access to the intracellular leaflet of organellar membranes including the ER, the site of PLs biosynthesis, and the outer envelope membrane of plastids, the site of galactolipid biosynthesis[98]. The lipidomic studies of leaf samples from plants under phosphate deprivation revealed a reduction of PC and PE, hence the hydrolysis of these lipid species catalyzed by NPC5 were included in the Plant Lipid Module[97]. Another event observed during Pi starvation is the extraplastidic accumulation of DGDG in mitochondria of Arabidopsis. The

transport of DGDG from plastid envelopes to mitochondria is therefore added to the Plant Lipid Module via MCSs as it is suggested to be dependent on contacts between these organelles[65,70,98–100].

The metabolic routes for the biosynthesis of a set of organic cofactors required in the synthesis of the different lipid moieties are also part of the model, which include the generation of coenzyme A (CoA), S-adenosyl methionine (SAM), FAD^+/FADH, NAD^+/NADH, $NADP^+$/NADPH, thiamine pyrophosphate, tetrahydrofolate (THF), adenosine triphosphate (ATP), cytidine triphosphate (CTP), guanosine triphosphate (GTP), uridine triphosphate (UTP), lipoamide, and nucleotide sugars (e.g. ADP-Glucose, GDP-Mannose, UDP-β-L-arabinopyranose, UDP-Glucose, UDP-α-D-galactose, UDP-6-sulfoquinovose, UDP-α-D-glucuronate, UDP-α-D-xylose). The allocation of transport reactions was based on extensive literature search to have confidence about location, stoichiometry, and transport mechanisms. Hence, a total of 11 extracellular transport reactions were added for the import of inorganic nutrients (e.g. Fe, H_2S, SO_4, NH_4, NO_3, PO_4^{3-}, Mg), other inorganic compounds (e.g. H_2O, H, CO_2), and photons; we also included 26 reactions modeling the movement of the aforementioned inorganic compounds across compartments. In addition, a total of 679 intracellular transport reactions were added to model the trafficking of compounds across compartments and ensure production of biomass, which include (i) 211 reactions for the transport of different lipid species and 356 reactions for the transport of lipidic precursors for the synthesis of cutin and the plasma membrane, (ii) 50 reactions for the transport of cofactors and their respective precursors, (iii) nine reactions for the transport of amino acids, (iv) 46 reactions for the transport of metabolic intermediates of central metabolism, and (v) seven reactions for transporting nucleotide sugars (see Supplementary Data 16). The modeling procedure for lipid metabolism also comprised the introduction of mathematical constructs as described by Sanchez *et al.*[101], whereby lipid classes were split into their basic components, and lipid pseudo-reactions, known as Split Lipids Into Measurable Entities (SLIMEr), which were defined to impose constraints on both lipid classes and acyl chain distributions. The implementation of SLIME consists on the following steps: (1) adding pseudo-metabolites to the model representing each specific backbone (one backbone for each lipid class, e.g. DG-backbone, TG-backbone, and so on) and each specific acyl chain (one for each FA species, e.g. C16:0-chain, C18:0-chain, and so on), besides a generic backbone and a generic acyl chain that groups all the backbones and acyl chains into a single entity, respectively; (2) adding a SLIME reaction for each specific lipid species, that consist of decomposing each lipid species into its basic components (i.e. backbone and acyl chains), that will later allow any of the specific lipids to form the generic lipid class; and (3) adding three new lipid pseudo-reactions to the model: (i) the backbone pseudo-reaction that pulls all backbone species previously created into a generic backbone and uses the corresponding experimental abundance data as stoichiometric coefficients; (ii) the analogous acyl chain pseudo-

reaction, for the acyl chains, parameterized with experimental data from FA methyl ester (FAME) analysis to create a generic acyl chain; and (iii) a reaction that merges the generic backbone and the generic acyl chain into a generic lipid that is later included in the biomass reaction.

This approach has proved to improve the accuracy of predictions related to the amounts of lipid species, the flexibility of the resulting lipid distributions and the computation of energy costs for lipid synthesis under different metabolic states[101]. Furthermore, the flux simulations in SLIME respects the experimentally determined acyl chain distribution, while avoiding over-constraining the model to only simulate one lipid distribution[101].

An additional step was added to the original SLIME implementation for all species belonging to the glycerolipids and glycerophospholipids categories, and the wax monoesters lipid class, which consisted of grouping the species of a determined lipid class according to the number of carbon atoms in the molecule (see Equations **1-3**), followed by grouping the above subgroups to form a single pseudo-metabolite (Equation **4**):

Step 1:

$$\mathrm{Rxn}_1\colon \mathrm{DG32\colon 0} + \mathrm{DG32\colon 1} + \mathrm{DG32\colon 2} + \cdots + \mathrm{DG32\colon X} \rightarrow DG32_{\mathrm{backbone}} \quad \textbf{(1)}$$

$$\mathrm{Rxn}_2\colon \mathrm{DG34\colon 0} + \mathrm{DG34\colon 1} + \mathrm{DG34\colon 2} + \cdots + \mathrm{DG34\colon X} \rightarrow DG34_{\mathrm{backbone}} \quad \textbf{(2)}$$

$$\mathrm{Rxn}_3\colon \mathrm{DG\ C}_{n1}\colon 0 + \mathrm{DG\ C}_{n2}\colon 1 + \mathrm{DG\ C}_{n3}\colon 2 + \cdots + \mathrm{DG\ C}_{ni}\colon \mathrm{X} \rightarrow DG\ \mathrm{C}_{n_{\mathrm{backbone}}} \quad \textbf{(3)}$$

where, DG stands for diacylglycerol; X indicates the number of double bounds in the lipid molecule; C_n represents the number of carbon atoms in the molecule; i is the i-th subspecies of the pool of lipids which have the same number of carbon atoms.

Step 2:

$$\mathrm{Rxn}_4\colon DG32_{\mathrm{backbone}} + DG34_{\mathrm{backbone}} + \cdots + DG\ \mathrm{C}_{n_{\mathrm{backbone}}} \rightarrow DG_{\mathrm{backbone}} \quad \textbf{(4)}$$

Demand reactions were added for several cofactors (e.g. CoA, $NADP^+$,CM[T]P, UTP, (R)-N6-dihydrolipoyl-L-lysyl-(protein) (DHL), (Fe-S)-cluster scaffold protein (Fe-S-SP)), because they were detected to participate in blocked reactions even when the respective metabolites are well connected in the metabolic network and were not identified as gap metabolites. The reason for this discrepancy was due to the "coenzyme pseudo-gap problem", occurring when the biosynthetic pathway for the *de novo* synthesis of a particular molecule is included in the reconstruction (as is our case), but there are no fluxes draining or degrading the said molecule produced by this pathway. Ponce-de-León *et al.*[102] described that these metabolites may be involved in conserved moieties, and in such cases, they will

be consumed and regenerated in a cyclic manner. As a consequence, they could not be detected as gap metabolites because they will participate in at least two active reactions. However, if the biosynthetic pathway for a coenzyme is included in a metabolic model, the net production of a coenzyme will not occur under steady state unless some flux consumes it[102]. Hence, the reactions involved in the biosynthesis pathway may become blocked. A common approach to solve this issue is to include the coenzyme-like metabolite into the biomass equation; however, as we do not have direct experimental measurements about these coenzyme concentrations in the biomass of all the Arabidopsis accessions, the alternative is to introduce an exchange flux that can drain the metabolite out of the system, which was done by introducing the demand reactions. We would like to note that the model includes reactions that are unbalanced in any metabolic network reconstruction[103], including the biomass reaction, 150 exchange reactions, 20 demand reactions (included for coenzymes), 11 import reactions and 1629 SLIMEr pseudo-reactions representing lipid formation.

Finally, we would like to point out that each of the lipid species included in the Plant Lipid Module is assigned information consisting of a code according to the LIPID MAPS nomenclature for each lipid class[104] (Supplementary Data 1), followed by the sum composition (C-atoms: double bond equivalents (DBE)), and the complete information at the structural level (i.e. TG 52:2 (16:0/18:1(9Z)/18:1(11Z))). For the structural information we note that for some sn-positional isomers (i.e., lipid species that present the same type of acyl chains, but they are at distinct locations along the lipid backbone (e.g. sn-1, sn-2, or sn-3)) that share the same origin (prokaryotic or eukaryotic route) and subcellular location, a single metabolite species was created as a representative for the pool of metabolites. This was done to prevent unnecessarily increase in the number of species in the model and thereby help to reduce the computational burden. Hence, the metabolite representing a pool of several sn-positional isomers is identified by separating the acyl chains with an underscore (C-atoms:DBE_C-atoms:DBE).

Supplementary Method 2. Integration of the lipid metabolic network into existing plant models

To test the functionality of the Plant Lipid Module, it was initially integrated into five different published models, the AraCore model[38], the Evidenced-Arabidopsis-Model[54], the GEM created in the Path2Models project and published by[105], the CAM diel model[106] and a medium scale model build for *Jatropha curcas*[107]. These model fulfilled the following requirements: (i) have COBRA model structure[108]; (ii) include metabolites with molecular neutral and/or charged formulas (when available), accompanied by the respective charges, the KEGG identifiers, and if possible, the ChEBI, and other identifiers; (iii) and were imported and exported (e.g. via COBRA Toolbox) without error or warning messages[109].

For the subsequent simulation procedures, the AraCore model hereinafter referred as the "Template Model" was used. Before proceeding with the merging of the models, several adjustments were carried out in the Template Model to guarantee the correct performance during the subsequent simulations. First, the KEGG identifiers were harmonized for three metabolites which are equivalent in the Plant Lipid Module, corresponding to beta-D-Fructose 1,6-bisphosphate (C05378) and beta-D-Fructose 6-phosphate (C05345) that are the isomers metabolized in the Glycolysis/Gluconeogenesis pathway; and beta-D-Glucose 6-phosphate (C01172) that is the isomer that act as intermediate in the pentose phosphate pathway. The KEGG identifiers were also added to a number of metabolites for which this information was missing.

To prevent the appearance of reactions with mass and charge imbalances due to differences between equivalent metabolites in the models, the molecular formula and/or metabolite charges were harmonized for the set of metabolites described next: (i) The metabolite dihydrolipoamide was assigned the molecular formula C14H26N2O2S2 corresponding to the metabolite (R)-N6-dihydrolipoyl-L-lysyl-[protein] that participates in the reaction EC:2.3.1.12 catalyzed by the component E2 (dihydrolipoamide acetyltransferase) that belongs to the pyruvate dehydrogenase complex (LTA3; https://www.uniprot.org/uniprot/Q0WQF7); (ii) Acetyldihydrolipoamide is generated as a product in the same reaction (EC:2.3.1.12), where (R)-N6-(S8-acetyldihydrolipoyl)-L-lysine residue has the molecular formula C16H28N2O3S2 assigned; (iii) Succinyldihydrolipoamide was assigned the molecular formula C18H30N2O5S2 corresponding to (R)-N6-(S8-succinyldihydrolipoyl)-L-lysine, according to the reaction EC:2.3.1.61 catalyzed by the component E2-1 (dihydrolipoamide succinyltransferase) of the 2-oxoglutarate dehydrogenase complex (At5g55070; https://www.uniprot.org/uniprot/Q9FLQ4); (iv) The reaction EC:1.9.3.1 transferred to EC:7.1.1.9 is catalyzed by cytochrome c oxidase, the last enzyme in the mitochondrial

electron transport chain, where the respective charges for [Fe(II)cytochrome c] and [Fe(III)cytochrome c] are +2 and +3, respectively (https://www.uniprot.org/uniprot/P93285). These charges are corrected in AraCore model since a -2 charge was assigned to both metabolites. A generic molecular formula (FeX) is also used for these metabolites to facilitate the mass balancing; (v) Aminomethyldihydrolipoylprotein participates in the reaction EC:1.4.4.2 catalyzed by the glycine decarboxylase or glycine cleavage system where (R)-N6-(S8-aminomethyldihydrolipoyl)-L-lysine residue has the molecular formula C15H30N3O2S2 assigned; (vi) The order of the elements in the molecular formula of acyl-carrier-protein (ACP) is adjusted alphabetically to HRS and the charge of the compound is changed from 0 to -1; (vii) The charge of Malonyl-ACP was adjusted to take into account the extra -1 charge contributed by ACP; (viii) The charge of the oxidized form of NAD+ was adjusted to be -1 since when this cofactor is oxidized the negative charge of one phosphate group is neutralized by the cation derived from the tetravalent state of the nitrogen atom of the nicotinamide group; (xi) The charge of the cytosolic NADH was originally set to -1, and was corrected to -2, since the reduced form of this cofactor has two phosphate groups and the nitrogen atom of the nicotinamide group has a trivalent state; (x) The charge of the cofactors NADP+/NADPH were also adjusted for the same reasons explained before for NAD+, hence the required adjustments are made to have an oxidized form with charge of -3 and the reduced form with charge equal to -4; (xi) The charge and molecular formula of the cofactor GDP is adjusted, since due it has two phosphate groups the charge corresponds to -3 and the corresponding formula is C10H12N5O11P2 (https://www.rhea-db.org/rhea/15753); (xii) The charge and formula of GMP are adjusted to -2 and C10H12N5O8P, respectively (https://www.uniprot.org/uniprot/P93757; https://www.uniprot.org/uniprot/Q9CAD1); (xiii) The charge and molecular formula of GTP are adjusted to -4 and C10H12N5O14P3, respectively (https://www.rhea-db.org/rhea/15753); (xiv) The charge and molecular formula of UDP are adjusted to -3 and C9H11N2O12P2, respectively (https://www.rhea-db.org/rhea/19929); (xv) The charge and molecular formula of 5-Amino-1-(5-Phospho-D-ribosyl)imidazole-4-carboxamide are adjusted to -2 and C9H13N4O8P (https://www.uniprot.org/uniprot/Q8RY94, https://www.rhea-db.org/rhea/23920); (xvi) The charge and molecular formula of 5-Aminoimidazole ribonucleotide are adjusted to -1 and C8H13N3O7P (https://www.rhea-db.org/rhea/10792, https://www.uniprot.org/uniprot/Q84TI2); (xvii) The charge and molecular formula of Acetyl-CoA are adjusted to -4 and C23H34N7O17P3S (https://www.rhea-db.org/rhea/16845, https://www.uniprot.org/uniprot/Q9LXS6); (xviii) The charge of hydrogen atoms are adjusted to be +1.

One of the metabolic intermediates that participates in the reactions EC:1.2.4.1/1.8.1.4/1.2.4.2 corresponds to a protein/enzyme N6-lipoyl-L-lysine residue. In the AraCore model there are two

metabolites which represent this intermediate, namely Lipoamide and Lipoylprotein. Since there is no net consumption of these metabolites in the model and to facilitate the integration of the Plant Lipid Module with the AraCore model, it is assumed that the N6-lipoyl-L-lysine corresponds to the same species derived from the lipoic acid metabolism generated in the mitochondria. Hence, the mitochondrial metabolites LPA and LPL in the AraCore model are integrated as one single metabolite corresponding to DLipoyl. Finally, a pseudo-metabolite named 'pho_loss' which accounts for the transference of photons was added to the photosystem reactions, in order to mass balance these reactions.

After performing these adjustments we implemented the “LipidModuleIntegration” procedure in MATLAB (R2022a, The Mathworks Inc.), interfacing with COBRA Toolbox v3.0[109], the set of MATLAB functions “Stoichiometry Tools” (https://www.mathworks.com/matlabcentral/fileexchange/29774-stoichiometry-tools), and the Gurobi Optimizer (version 8.0.1), to integrate the two models and obtain a merged model containing all the reactions of the Plant Lipid Module. Briefly, the compartments information is retrieved from both models and compared to identify common compartments, followed by the harmonization of their respective abbreviations in the metabolites list. The software also identifies the existence of the plastid compartment in the Template model, and when present, a verification procedure is carried out to identify the presence of the light reactions to guarantee the production of ATP and reducing power. Otherwise, a message is displayed asking the user for their inclusion in the Template Model. Next, a graphical user interface is launched where the compartments of the Template Model are displayed in a drop-down list (red arrow on the right-hand side of the User Interface shown in Supplementary Fig. 9), along with a table containing the respective abbreviations and names of the compartments of the Plant Lipid Module for which an equivalent compartment was not found in the Template Model (left-hand side of the User Interface shown in Supplementary Fig. 9), giving the possibility to the user to carry out the pairing manually. This feature of the software is particularly helpful in the event that compartments have ambiguous names, hence are not correctly identified by the software.

Before the integration takes place, the software performs a check up to identify the metabolites which are identical in the two models by comparing the available metabolite identifiers, charge and molecular formulas, with the purpose of harmonizing their usage in the merged model and prevent the presence of duplicated metabolites. The next step is the integration of the metabolites of the Plant Lipid Module into the Template Model, by means of which the respective information of molecular formula, charge, KEGG, PubChem, CheBI, Lipid Maps and International Chemical Identifier (InChI) identifiers is added. The last step performed by the software is adding the reactions of the Plant Lipid

Module to the Template Model, followed by the execution of an additional step for removing the duplicated reactions by checking the stoichiometric matrix, and detecting the columns of the matrix that are identical up to scalar multiplication[109]. The Template Model with the integrated Plant Lipid Module is printed out, besides the list of paired and unpaired compartments, and the list of new metabolites added to the Template Model.

The merged model created for the simulation purposes using the AraCore model as template, required further adjustments to ensure its correct functionality and to avoid the emergence of errors or unexpected results, which briefly consisted in the elimination of metabolites that were not participating in any reaction, adjustment of mass- and charge-imbalanced reactions. The reversibility of the removed reactions was verified to ensure that it will remain according to how it was assigned in the Plant Lipid Module reactions. The newly added-reactions from the Plant Lipid Module were checked to identify mass- and charge-imbalances derived from differences in the charged formulas of the equivalent metabolites contained in the models.

The Plant Lipid Module and the "LipidModuleIntegration" tool are available online in Zenodo (DOI: 10.5281/zenodo.8179057)[110] and GitHub (https://github.com/marce2336/PlantLipidModule.

Supplementary Method 3. Integrating quantitative information of biomass components

The biomass reactions account for all major cell components, namely cell wall, proteins, lipids, soluble metabolites, starch, DNA and RNA. These components are incorporated via representative metabolites or precursors as explained in detail next:

Proteins

the protein-bound amino acids were considered as representative metabolites for cell protein. To estimate the amount (µmol g^{-1} DW) of each amino acid in the biomass the following steps were followed:

First, a list including abundance data for 4190 proteins identified in leaves of 4.5 - 5 weeks-old soil grown *Arabidopsis thaliana* Heynl. wild-type (Columbia-0) (hereinafter referred as Col-0), under 14-h light photoperiod and 100 µE m^{-2} s^{-1} light intensity, was retrieved from PAXdb: Protein Abundance Database (https://pax-db.org/species/3702)[111,112]. This list was matched with the proteins detected in the proteome analysis referred below, to estimate the proportion of the most abundant proteins that were included in the calculations. According to these estimations, a number of proteins accounting around 80% of the most abundant proteins were included.

The amino acid composition was first estimated for leaves of Arabidopsis plants grown under standard conditions. The standard conditions here refer to 3–5-week-old Arabidopsis plants, Col-0 ecotype, grown under a photoperiod of 12-16h light, temperature ranging among 20-22°C and relative humidity ranging 60-75%. Hence, publications were reviewed for proteomics analysis of aerial organs of Arabidopsis plants that meet the searching criteria. The proteomic data published by[113] was used, where the Arabidopsis wild type Col-0 plants were grown on soil under continuous white light conditions at 22 °C. The leaf samples were taken from juvenile and adult rosette leaves, harvested at the same time point from 22 days-old plants before bolting. Protein abundance estimation was based on corrected TMT (tandem mass tags) reporter intensities.

The length of the amino acids chain and its respective amino acids sequence was retrieved for each protein from the Uniprot database and used to estimate the amino acid composition for each protein identified in the proteomic analysis of Arabidopsis leaves under standard conditions. Only proteins with curator-evaluated data were considered (The UniProt Consortium 2021).

Since it is difficult to find condition-specific proteomics data sets for plants subjected to the stress conditions resembling the exact experimental setup, we focused on finding publications with

conditions as close as possible to those used in the present study in terms of plant ecotype, temperature, illumination, age of plants and the specific stress condition, namely extended darkness. It was possible to obtain proteomics data for 3-weeks-old Arabidopsis Col-0 plants grown under extended darkness during 8 h at 21 °C[114]. The list of proteins identified in the stress condition were matched against the quantitative proteomic data of Arabidopsis under standard conditions. When a common protein was identified, the protein abundance fraction was adjusted according to the values of the ratios (dark- vs. light-cultivated plants). The proteins that were not identified in the dark-grown plants or for which no change was reported against the control samples, were assumed to have the same abundance value as in the standard condition.

The data for total protein content in Arabidopsis plants subjected to the said stress conditions was also searched in the literature. In the work published by[115] the total protein content of Arabidopsis ecotype Col-0, grown at 20°C was measured after 3 and 6 days of cultivating the plants under darkness, with values of 10.35 and 9.31 mg g^{-1} FW, respectively. The conversion of FW into DW was conducted by assuming a dry weight content under conditions of optimal growth of 0.088 (g DW g^{-1} FW), giving as a result 117.59 and 105.79 mg g^{-1} DW, respectively[38,116]. For Arabidopsis plants under standard conditions a total protein content of 243.79 mg g^{-1} DW was assumed[38,117].

The protein abundance fractions estimated for the standard and stress conditions together with the total protein data previously mentioned was used to assign the fractional amount of the protein-bound amino acids of Arabidopsis leaf. Since there is no accession-specific data for the protein-bound amino acids, it is assumed that the values estimated for the ecotype Col-0 under the stress conditions is the same for all the accessions.

The ultimate amino acid fraction in the biomass composition is the combination of free and protein-bound amino acids. Hence, the soluble intracellular metabolites were measured by GC-MS in a population comprised of 284 Arabidopsis natural accessions belonging to the HapMap panel[118], to maximize diversity and minimize redundancy and close family relatedness[119]. The plants were grown during six weeks (16-h light; 21/16 °C, day and night, respectively; light intensity 150 μE m^{-2} sec^{-1}; relative humidity of 75%) and then transferred to continuous darkness either for 3 or 6 days. Between 3 and 6 plants from each accession were collected for each condition tested. The measured metabolites consisted of the relative abundance for free amino acids, organic acids, sugars, and several intermediates of primary and secondary metabolism. To determine the metabolites content, the absolute concentration levels of GC-MS/MS data for Arabidopsis (Col-0) from[117] were adjusted by multiplying by the respective ratios (dark- vs. light-grown plants) calculated from the relative

abundance data. The content of free amino acids was finally added to the previously estimated protein-bound amino acids.

Starch and sugars

Quantitative data for starch accumulation in Arabidopsis plants subjected to extended darkness (i.e. 3 and 6 days) was obtained from the publication of[115], and is presented as hexose equivalents. The data for organic acids (succinate, fumarate, malate), sugars (i.e. fructose, glucose, sucrose, trehalose) and several intermediates of primary and secondary metabolism (i.e. GABA, ornithine, urea) was obtained and handled as explained previously for free amino acids. In brief, the absolute concentration levels of Arabidopsis (Col-0) obtained from[117] were adjusted by multiplying by the corresponding ratios (dark- vs. light-grown plants) calculated from the relative abundance data.

Starch is represented by amylose, where a Glc dimer precursor has been incorporated in the model to account for this biomass component.

Cell wall and nucleic acids

To account for cell wall and nucleic acids as biomass components the same procedure was followed as previous report [38]. The cell wall is represented by cellulose, which was incorporated in the biomass reaction as a glucose (Glc) dimer precursor. Since it is not possible to find condition- and/or accession-specific experimental information about the quantification of cell wall and nucleic acids, these components are incorporated in a condition-unspecific manner. The cell wall fraction used for calculations corresponded to 118 µg cellulose mg^{-1} DW according to values published by[120] that was later converted into 363.88 µmol Glc units of cellulose g^{-1} DW by considering a molar mass of anhydroglucose, 162.14 g mol^{-1} [121]. The nucleotides are considered as representatives for DNA and RNA. For the respective calculations, a DNA content of 0.092 mg DNA g^{-1} FW measured in Arabidopsis leaves was taken from[122] which was also assumed to be an appropriate level for RNA. Based on the DNA and cDNA sequence of Arabidopsis, the nucleotide fraction was determined, which subsequently was converted into the respective nucleotide levels by means of the molar mass of each nucleotide.

Lipids

In the previous publication [38], palmitic acid was considered as a representative FA and malonyl-acyl carrier protein (M-ACP) was incorporated as the corresponding precursor. In the same publication, the total lipid content was assumed to correspond to the total FAs amounting to 3.3 mg g^{-1} FW in leaves of wild type Arabidopsis[123]. In the Plant Lipid Module an explicit representation was made for

the lipid reactions generating structural and storage lipids, besides the incorporation of SLIME reactions according to[101], which allow the representation of lipids as biomass requirements, taking into consideration the numerous possible combinations of individual lipid species, hence allowing the incorporation of commonly available experimental data as constraint of lipid classes and acyl chain distributions.

Since it is not possible to obtain condition- and accession-specific quantitative data for all the lipid species that may be present in a determined sample, a search in the literature was made to retrieve data for the absolute content of lipids in leaves of Arabidopsis plants grown under standard conditions. From the publication of[124] it was possible to obtain values (µmol g^{-1} FW) for 125 lipid species distributed in various classes, namely PC, PE, PG, PI, PS, DG, TG, free FAs, CL, MGDG, and DGDG, measured in 4-week-old plant rosette leaves of Arabidopsis ecotype Col-0 grown on soil under 16-h light/8-h dark cycle, temperature of 22°C, and relative humidity of 70%. Data for sphingolipid composition (nmol g^{-1} DW) which include species of ceramide, 2-hydroxyceramide, glucosylceramide, and glycosylinositolphosphoylceramide, were obtained from[12] for Arabidopsis leaf tissue at 5–6 weeks of age. Data for wax measured in stem and leaf tissue of 6-week-old Arabidopsis plants were obtained from[125], which include FFAs, aldehydes, primary alcohols, alkanes, and ketones. Since the concentration was reported as µg/dm^2, units were converted into µg g^{-1} DW, by using a conversion factor of 0.1664 g DW/dm^2 calculated from data for total leaf area and dry biomass content published by[126] for 4 and 6 week-old Col-0 Arabidopsis plants. Cutin monomer composition of leaf tissues from 5-week-old Arabidopsis plants was obtained from[12,127] which includes FAs, ω-hydroxy FAs and α,ω-Dicarboxylic acids. Since lipid species content is expressed as µg/dm^2, the same procedure aforementioned was used to convert the units into µg g^{-1} DW. Finally, total chlorophyll content was obtained from the publication of[128], for light-grown plants (1.52 mg g^{-1} FW) and plants subjected to extended darkness for three (1.39 mg g^{-1} FW) and six (1.28 mg g^{-1} FW) days.

A targeted lipid profiling analysis by LC–MS was performed as described below in the population of Arabidopsis natural accessions panel described earlier. The experimental setup for the light-grown plants resembled those used for the cultivation of plants grown under standard conditions from which the quantitative data was obtained. The lipidomic analysis allowed obtaining the relative abundances for 138 lipid species of the following classes: DG, DGDG, MGDG, sulfoquinovosyldiacylglycerol (SQDG), PC, PE, PG and TG. The relative abundance data were used in turn to calculate the ratios among Dark- vs. Light-grown plants. To obtain the lipids content for the accessions, the absolute content retrieved for leaves of Arabidopsis plants grown under standard conditions is adjusted for

each accession by multiplying the quantitative data by the respective abundance ratios. There were several lipid species that were detected in the light-grown plants but not in the dark-grown plants, and vice versa therefore, to facilitate the calculations it was assumed that these species were under the detection limit, hence it was assumed that their abundance was equal to 1/5 of the minimum positive abundance value of their corresponding variables.

Due to the lack of accession- and condition-specific quantitative data for several lipid classes, namely free FAs, CLs, sphingolipids, wax and cutin monomers, whose summed content is roughly equivalent to 0.7% of the total dry biomass content of Arabidopsis leaves[12], it is assumed that their content remain equal to the samples grown under standard conditions.

Experimental setup for the targeted lipidomic analysis

The Arabidopsis accessions were cultivated and handled as described above in the 'Proteins' subsection. The extraction was carried out using 100 mg of leaf sample from each of the accessions, and the analysis was performed as described[129]. In brief, the dried organic phase was measured using Waters Acquity ultra471 performance liquid chromatography system (Waters, http://www.waters.com) coupled to Fourier 472 transform mass spectrometry (UPLC–FT–MS) in positive ionization mode. Analysis and processing of raw MS data were done with REFINER MS® 10.0 (GeneData, http://www.genedata.com). Workflow included peak detection, retention time alignment and removal of chemical noise and isotopic peaks from the MS data. Obtained mass features characterized by specific peak ID, retention time, m/z values and intensity were further processed using custom scripts in R (R Core Team, 2019). Clusters with mean of signal intensities lower than 50000 were removed and only peaks present in at least 70% of the samples were kept for further analysis. Peak intensities were normalized by the day of measurement and median. After that, obtained molecular features were queried against a lipid database for annotation. The in-house database used includes 219 lipid species of the following classes: DGs, DGDGs, MGDGs, PCs, PEs and TGs. This was carried out by comparing their retention times and exact masses against those of the reference compounds, allowing maximal deviations of 0.1 minutes and 10 ppm. Identified lipids were confirmed by manual verification of the chromatograms using XCalibur (Version 3.0, Thermo-Fisher, Bremen, Germany).

Supplementary Method 4. Creation of accession- and/or condition-specific biomass reactions

For the generation of biomass reactions which are either condition- or accession-specific, we created a set of scripts which retrieve the experimental data for the desired Arabidopsis accession and growth condition and calculates the stoichiometric coefficients for each biomass component. Here we would like to emphasize that the experimental lipid data to be provided for the analysis can be of two types: (i) abundance data for each lipid class and for the total FAs (e.g. FAME analysis) or (ii) abundance data provided for each lipid species measured by means of a lipidomics analysis. For the first case, the abundance data for the lipid classes and the FAs can be used to directly assign the coefficients for the backbone- and chain- pseudo reactions, respectively. In the second case, a different procedure is implemented to estimate the abundance for all the acyl chains included in the acyl chain pseudo-reaction. Before carrying out the estimations, the absolute data for the measured lipids (g g^{-1}DW) must be provided where each lipid species must be identified by their corresponding lipid class abbreviation (see Supplementary Data 1), followed by the sum composition, and the structural details in case this last information is available, otherwise the sum composition data is sufficient. The designed algorithm executes the steps described next: (i) each lipid species is assigned its respective code according to the LIPID MAPS nomenclature as shown in Supplementary Data 1[104]; (ii) the list of metabolites (chains and backbones) participating in the lipid pseudo-reactions is retrieved from the model; (iii) each of the lipid species is traced back into the model to identify the species matching the sum composition and structural information provided or in case that only the sum composition is available, the algorithm identifies all the matching mass isomers; (iv) when a matching species is identified in the model, the respective acyl chains are identified and stored in a separate list where their abundance is estimated according to their molar ratio in the lipid molecule and the absolute abundance provided for the said lipid species; (v) once this step is performed for all lipids, the abundances for all common backbones and chains are summed up and are later used as the respective stoichiometric coefficients for the lipid pseudo-reactions.

Here we would like to emphasize that depending on the methodology and the equipment used, lipidomic analyzes can generate information on the lipid species present in a sample only at the sum composition level, or in addition to the latter, it can also provide details at the structural level. If data is available only at the sum composition level, the algorithm searches for all the species present in the lipid module that have the same sum composition and from this makes an estimate of the FAs

distribution. Consequently, it is assumed that the pool of a given lipid is made up of species that share the same molecular mass which are also assumed to be present in the total pool in the same proportion, but that may differ in the composition and stereospecific location of the FAs. In the case that information is provided at the structural level, this is used to identify the exact compound in the model.

Supplementary Method 5. Identification of synthetic lethal gene sets

To identify the combinations of genes, which when deleted abolish growth, we implemented the Fast-SL algorithm using the code available for MATLAB (R2022a)[130], and that was interfaced with the COBRA Toolbox v3.0[109], and the Gurobi Optimizer (version 8.0.1). Hence, after the integration of the Plant Lipid Module in the AraCore model, we estimated the biomass in optimal growth conditions ($max\ v_{bio}$) under the constraints of (i) steady-state of the model; (ii) lower and upper flux capacities (i.e., bounds); (ii) biomass reaction under standard conditions ($v_{bio,Col0}$), (vi) the bound on the ratio between the carboxylation and oxygenation reactions catalyzed by RuBisCO was set to 2.88[131], and (v) the stoichiometric coefficients for chain- and backbone-SLIME pseudo reactions were assigned according to[101] by implementing a custom script (see Creation of accession- and/or condition-specific biomass reactions):

$$\max v_{\text{bio}}$$

s.t.

$$\mathbf{N} \cdot v = 0, \quad \mathbf{(5)}$$

$$\forall i, 1 \leq i \leq n,\ \alpha_i\ \leq v_i \leq \beta_i,$$

$$v_{\text{carboxylation}} = 2.88 v_{\text{oxygenation}}$$

where α_i and β_i denote the generic lower and upper flux boundaries (−1000 and 1000, respectively, for reversible reactions and 0 and 1000, respectively, for irreversible reactions). Next, we constrained the model with the $max\ v_{bio}$ value and proceed to compute a reference flux distribution (v^{Col0}) via pFBA:

$$\text{min.} \sum \left| v_i^{\text{Col0}} \right|$$

s.t.

$$\mathbf{N} \cdot v = 0, \quad \mathbf{(6)}$$

$$v_{\text{bio}} = v_{\text{bio,Col0}}$$

$$\forall i, 1 \leq i \leq n,\ \alpha_i\ \leq v_i \leq \beta_i,$$

$$v_{\text{carboxylation}} = 2.88 v_{\text{oxygenation}}$$

Prior to the implementation of the Fast-SL algorithm, we verified that carrying out pFBA together with the set of constraints described above (see Equation **5**) were sufficient to substantially reduce the solution space and thereby obtain a unique solution. Hence, we performed a flux variability analysis (FVA) with pFBA. The FVA was conducted at optimum biomass (the biomass reaction was constrained with the maximum-predicted growth rate) and minimizing the flux through gene associated reactions by holding minimum network flux constant. The latter was done by first minimizing the absolute value of flux through all gene-associated reactions via pFBA, and next using this flux to constrain the upper bound for the summed network flux. Upon comparing the flux distributions (Supplementary Data 17), we found that the difference among the minimum and maximum flux values for 79% of the reactions that carry flux is close to zero (< 0.35 mmol g-1DW). The remaining reactions (21%) show a difference that ranges from 3 mmol g^{-1}DW up to the maximum value set for the upper bound (1000), from which 65% correspond to reversible reactions, which is fully justified. In the calculated FVA flux distributions, there was a group of reactions not carrying flux. For 60% of these reactions, the inactivity is condition-specific, which means that they are used in the simulation of lipid remodeling events taking place under different stress conditions. The remaining 40% inactive reactions are producing different lipid species and their intermediates which were not included in the biomass reactions, due to the absence of quantitative information for the condition simulated. Updating the biomass reaction in different scenarios (e.g. species, organs) will allow further activation of these reactions.

Once we verified that the implementation of the pFBA in a two-step procedure yields an evident reduction of the solution space, we continued with the computation of the sets of synthetic lethal genes. For this, the Fast-SL algorithm uses v^{Col0} to select the set of reactions that carry a non-zero flux (v_{nz}^{Col0}). At the same time, the reactions not carrying flux ($v^{Col0} - v_{nz}^{Col0}$) are excluded from the analysis, as they are regarded as not essential for the growth of the organism, and hence the search space for the synthetic lethals is reduced[130]. For the lethality analysis, we also excluded demand, exchange, maintenance, and the SLIME reactions, since these are regarded as pseudo-reactions and do not have GPR rules assigned[130]. The synthetic gene lethality analysis was then carried out as described [130], computing the sets of synthetic single lethal genes and lethal gene pairs. A gene was identified as essential (lethal) when biomass flux was abolished upon its deletion from the model. This analysis is done taking into account the presence of isoenzymes and enzyme complexes[130]. The set of non-lethal genes was obtained by removing the genes that were identified as lethal from the list of model genes. The accuracy of the results was verified using information on Arabidopsis mutants available on the SeedGenes Project[132] and ARALIPID website[12]. The data were filtered, discarding mutants that lacked the description of the resulting phenotype, as well as mutants corresponding to

genes that were not included in the model. The phenotype information of each mutant list was contrasted with the single lethal and non-lethal gene sets, and the proportion in which the phenotype predicted in the simulation was confirmed by the experimental data was determined for each case.

Supplementary Method 6. Condition-specific flux distributions for Arabidopsis accessions

For the simulation of the extended darkness conditions we used a similar approach to[133] for the generation of a diel flux balance model where the light and dark phases are simulated simultaneously in a single optimization problem. For the construction of the light/dark-model, the previously created Template Model (see Integration of the lipid metabolic network into existing plant models) was duplicated, and the reactions were differentiated with the _light or _dark suffix, and the metabolites in turn were labeled with 1 or 2 after the compartmentation suffix, which stands for light and dark, respectively. Next, we created transport reactions across phases that were identified by adding the suffix _LightDark to the reaction names, to model the traffic of a set of sugars (glucose, fructose, sucrose)[134], carboxylic acids (malate, fumarate, citrate)[134–136], and amino acids[137] towards the vacuole as an intermediate storage step between phases. The reactions are assumed to be irreversible for the transport of metabolites across phases given that the plants were subjected to extended darkness conditions. In addition, the extended dark condition implies that there is only a finite supply of metabolites that were stored during the light phase, hence the upper limits of transport reactions across phases were constrained to the fluxes leading to the accumulation of the respective metabolites under light conditions (v_{light}^{Col0}).

Since we made the assumption that each phase (i.e. light or dark) was in a pseudo-steady state, to allow the modeling procedure as a single optimization problem, the photoautotrophic phase was specified by allowing a photon influx, while for the heterotrophic metabolism the photon influx was set to zero. The import of oxygen, carbon dioxide, water and inorganic ions was allowed under the two phases. Other biomass compounds such as cellulose and nucleotides are assumed to remain constant during the light-dark phases since there are no accession- and condition-specific experimental data available.

On the other hand, FA synthesis is negligible in the dark[138–140]. In addition, there is evidence that free FAs levels (FFAs) in wild type plants remain low and unchanged under dark treatment[138]. For these reasons, it is assumed that most lipid classes are produced under light conditions, hence levels for several lipid classes including structural lipids and their precursors are assumed to remain constant during darkness, which include CL, PA, LPA, LPC, sphingolipids, hydrocarbons, oxo FAs, fatty alcohols, dicarboxylic acids, fatty aldehydes, and sterols. Chlorophyll is also assumed to be synthesized only under light conditions. There is another group of lipids whose levels changed across the light-dark phase according to the experimental measurements, which correspond to PC, PE, PG,

PI, PS, DG, TG, MGDG, DGDG, SQDG. For these lipids, it is assumed that the pools accumulated under light conditions are maintained until the darkness starts, from which point they are subjected to degradation/remodeling processes. To make available the lipids that were accumulated during the light phase to the model under the dark conditions, we added transport reactions of lipids across the phases without changing the subcellular location of the metabolites.

Finally, the accession-specific flux distributions were computed via pFBA, imposing the same constraints as described in Supplementary Method 5, except for the biomass reaction and the chain- and backbone-SLIME pseudo reactions whose respective stoichiometric coefficients were calculated from the values obtained from measurements performed for sugars, organic acids, amino acids, and lipids, for each accession subjected to extended darkness for three and six days, as explained in Supplementary Method 3 and 4.

Supplementary Method 7. Validation of the flux distribution results obtained under extended darkness

The validity of the results of the simulations obtained via pFBA under extended darkness conditions was verified by comparing the $\log_2$ FC values of the flux distributions with the $\log_2$ FC values for the transcripts measured under comparable environmental conditions.

The calculation of the flux distributions via pFBA, and the construction of condition-specific biomass reactions were performed according to the procedure described above (see Supplementary Method 6), with the exception of quantitative data for soluble metabolites and lipids that were measured in Arabidopsis ecotype Columbia-0 plants under control conditions and a period of 21 hours of extended darkness (21h-XD)[141]. Two sets of transcriptomics data for plants under 21h-XD were obtained from[141] and[142], and processed first by calculating the mean of the biological replicates, and then assigning the abundance of the transcripts for each enzyme included in the model according to the GPR rules, considering the presence of isoenzymes and protein complexes[143], followed by the calculation of $\log_2$ FC. The validity of the obtained flux distributions was evaluated by comparing the increase/decrease in $\log_2$ FC for the fluxes and transcripts for each enzyme and determining the concordance of the sign change for each case.

Supplementary Method 8. Identification of candidate genes that modulate leaf lipid metabolism

For this analysis, we made use of the flux distributions calculated for Arabidopsis accessions under extended darkness (see Condition-specific flux distributions for Arabidopsis accessions), where the fluxes were regarded as traits. For each flux, the R package rMVP (R version 4.2.1.) was used for genome-wide association studies (GWAS)[144]. A total of 1.329.408 SNPs from imputation of 1.001 Arabidopsis Genomes and Regional Mapping (RegMap) panel were tested, after filtering with minor allele frequency >0.05[145]. The GWAS linear mixed model considering both kinship and population structure was applied. The first three principal components were considered to represent population structure. The significant threshold was set to 1/n, where n is the number of SNPs. The significant SNPs within an interval of 20 kb were considered as one and all genes in the interval of the most significant SNPs were considered as the candidate genes.

The lists of candidates obtained were validated using information from GPR rules, and from transcription factors (TFs) and gene expression databases. Hence, from the GPR rules, the list of genes associated to each reaction flux was retrieved. Then, each gene was mapped to the list of candidate genes obtained for the corresponding trait. The mapping process of the candidate genes was later extended to the GPR rules associated to the reactions immediately up- and down-stream of each of the fluxes for which a list of candidates was available. The next step consisted in building a list with the TFs contained in the AthaMap database[146], that was used to select the candidate genes coding for TFs. Since the list of TF-encoding candidates was substantially large, we selected only those obtained for lipid-related reactions. For each TF-encoding candidate, the list of genes with potential binding sites was obtained[146]. The latter was mapped to the GPR rules of the underlying reaction flux. We further examined the TF-encoding candidates with DNA-binding domains for the genes of the corresponding GPR rules, by reviewing their expression profiles. For this, we searched on TRAVA database[3] the expression levels of genes in whole leaves. The read counts were normalized by applying the median-of-ratio method, followed by dividing by the maximum value of expression level, so all values vary from 0 to 1[3]. The candidates were classified into four categories according to their level of expression: (0) not expressed, (<0.3) low, (0.3≤ ≤0.6) intermediate, and (>0.6) high.

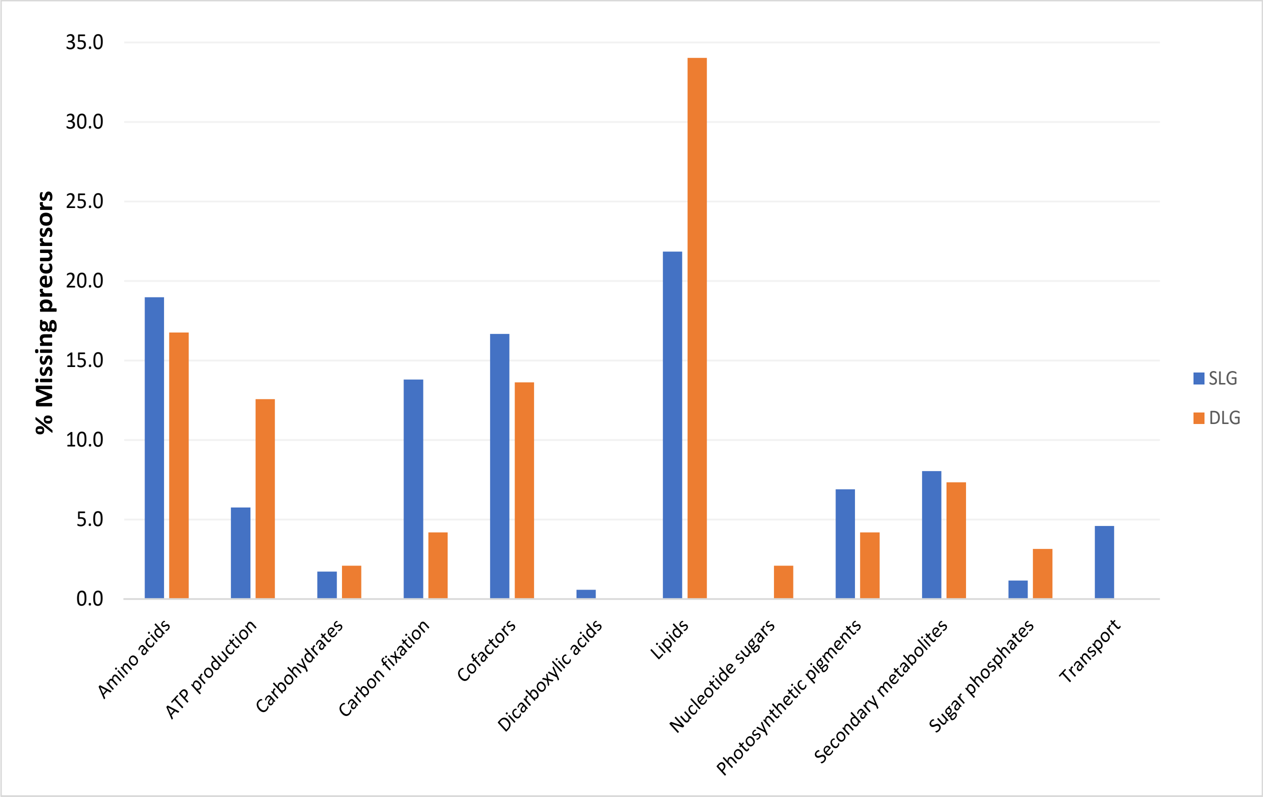

Supplementary Figure 1. **Impact of gene mutation on the synthesis of biomass precursors presented as percentage of missing precursors.** DLG, double-lethal genes; SLG, single-lethal genes. Source data are provided as a Source Data file.

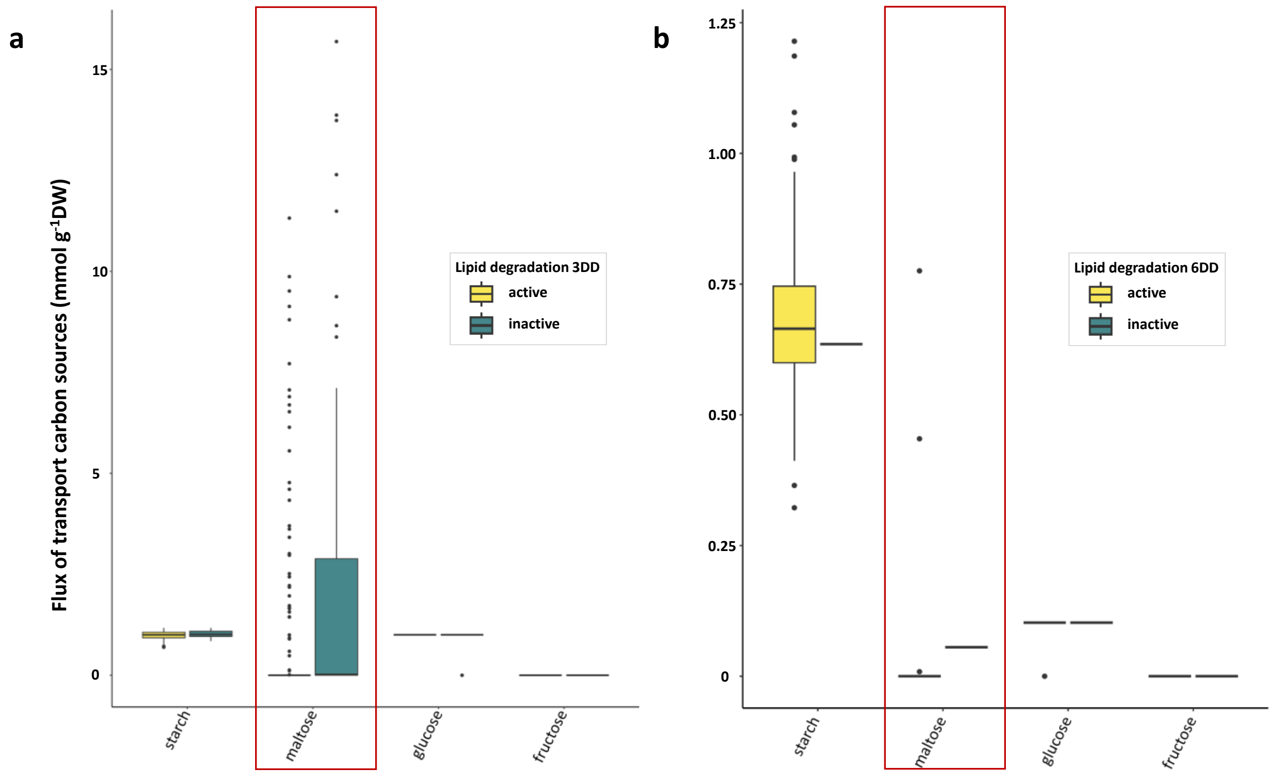

Supplementary Figure 2. **Rates (mmol g^{-1}DW) of transport for starch, maltose, glucose, and fructose, were computed via pFBA.** The metabolites accumulated during the photoautotrophic phase, subsequently serve as carbon sources after exposure of Arabidopsis accessions to periods of **(a)** 3 and **(b)** 6 days of extended darkness. The accessions were classified according to the predicted state of activity (active/inactive) of their lipid degradation pathways. In Arabidopsis accessions with inactive lipid degradation routes, the rates of consumption of maltose are predicted to be higher compared to accessions in which lipids are predicted to be degraded. In addition to maltose, the consumption of other storage compounds, such as starch and glucose, is also expected, although the consumption rates of the latter are comparable for both groups of accessions. The data to build the figure in the left panel were scaled by mean centering to improve the visualization. Source data are provided as a Source Data file.

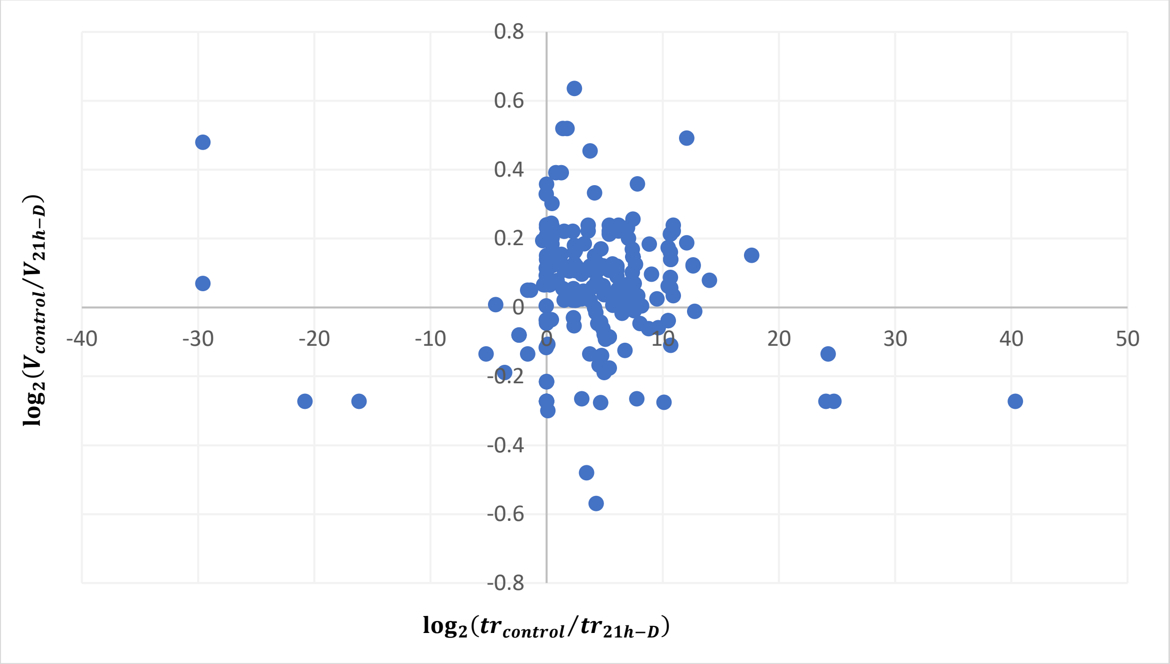

Supplementary Figure 3. Scatter plot of log 2=fold change for flux rates (V) and transcripts abundance (tr) for Arabidopsis Columbia-0 under control and extended darkness for 21 hours (21h-D). The data sets for transcripts abundance used for the calculations were obtained from the publication of Caldana *et al.*[141]. Source data are provided as a Source Data file.

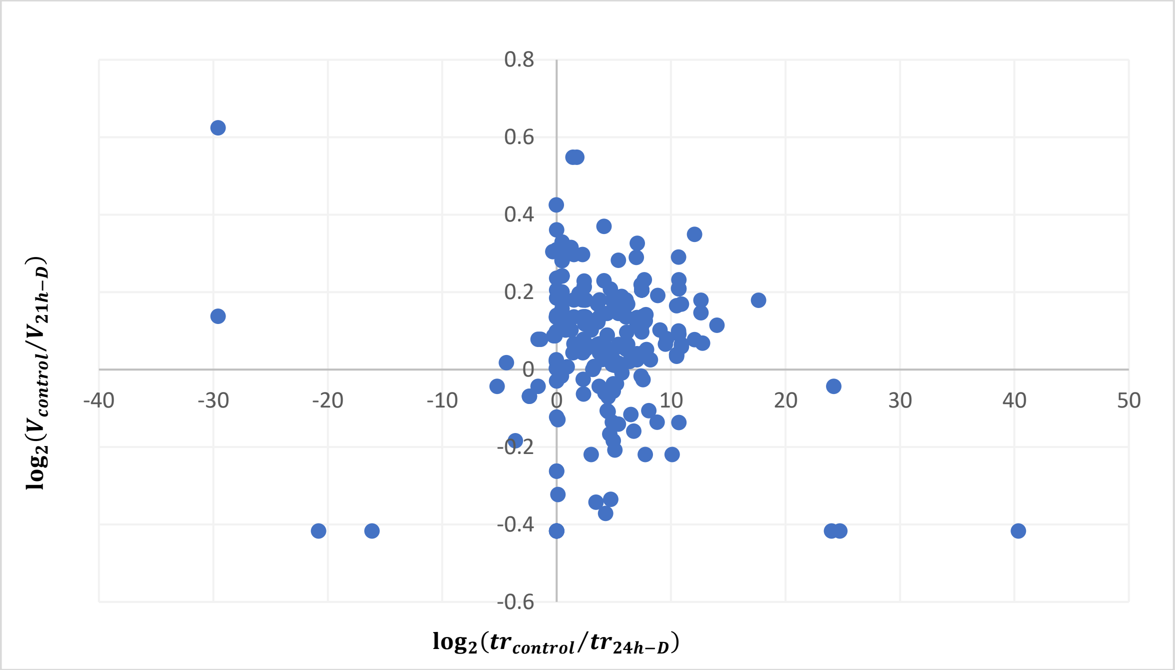

Supplementary Figure 4. Scatter plot of log 2-fold change for flux rates (V) and transcripts abundance (tr) for Arabidopsis Columbia-0 under control and extended darkness for 24 hours (24h-D). The data sets for transcripts abundance used for the calculations were obtained from the publication of Usadel *et al*.[142]. Source data are provided as a Source Data file.

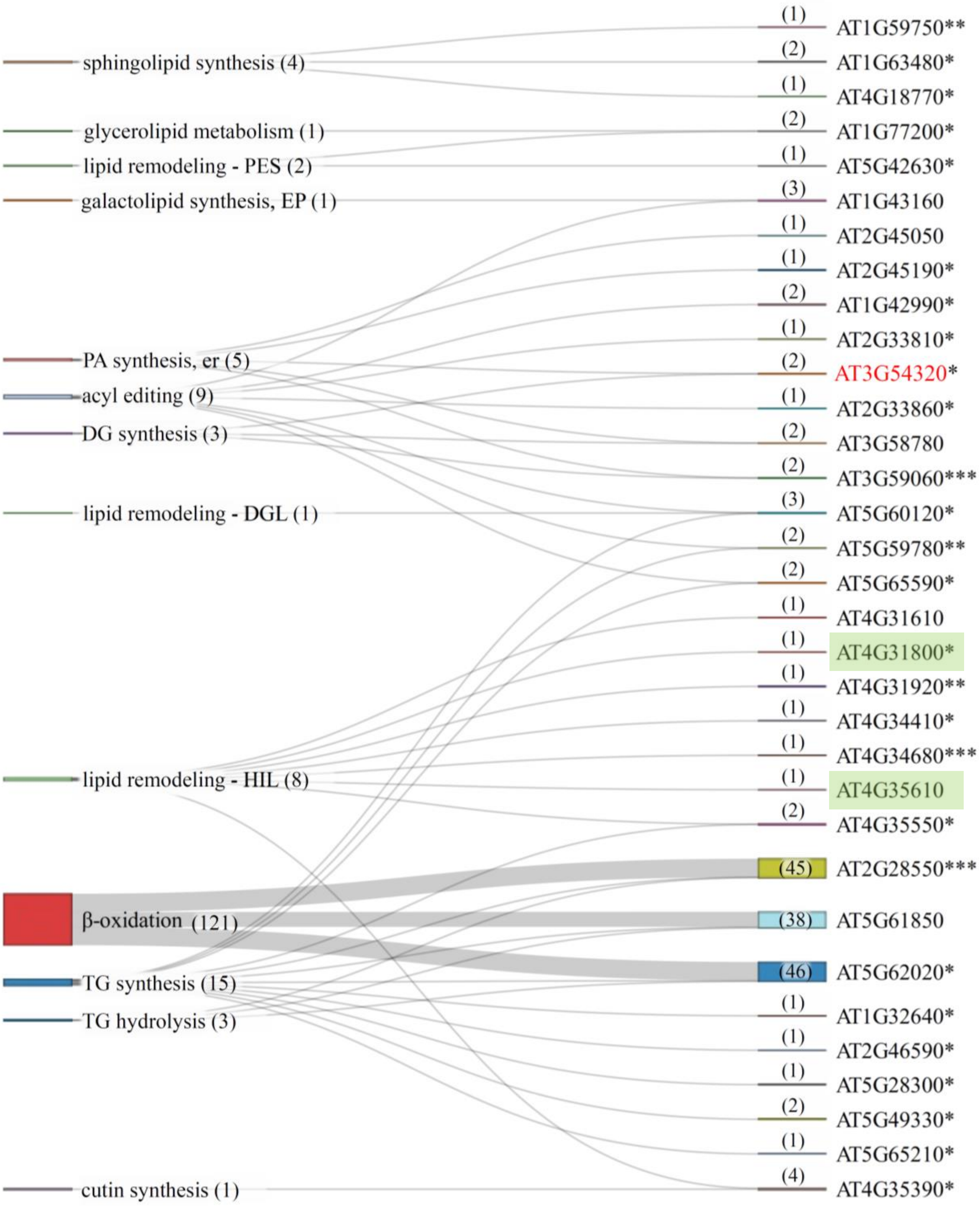

Supplementary Figure 5. **Candidate genes identified for Arabidopsis accessions under 3 days of darkness.** The diagram includes only the candidates that encode transcription factors (right) that contain DNA-binding domains for the genes of the GPR rules for which the candidate was identified. Traits are grouped by the metabolic pathway to which they belong (left). Source data are provided as a Source Data file.

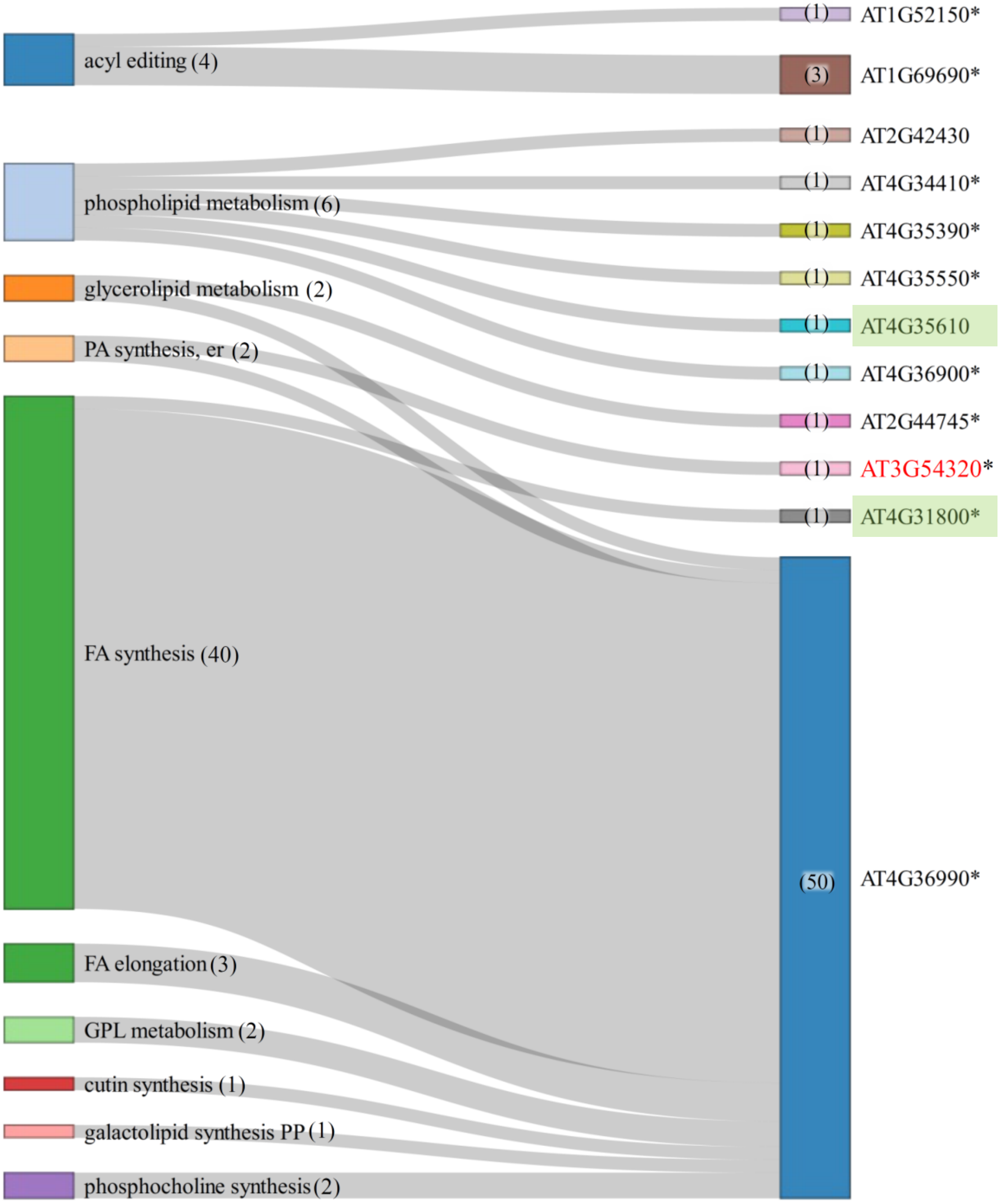

Supplementary Figure 6. Candidate genes identified for control samples of Arabidopsis accessions under 3 days of darkness. The diagram includes only the candidates that encode transcription factors (right) that contain DNA-binding domains for the genes of the GPR rules for which the candidate was identified. Traits are grouped by the metabolic pathway to which they belong (left). Source data are provided as a Source Data file.

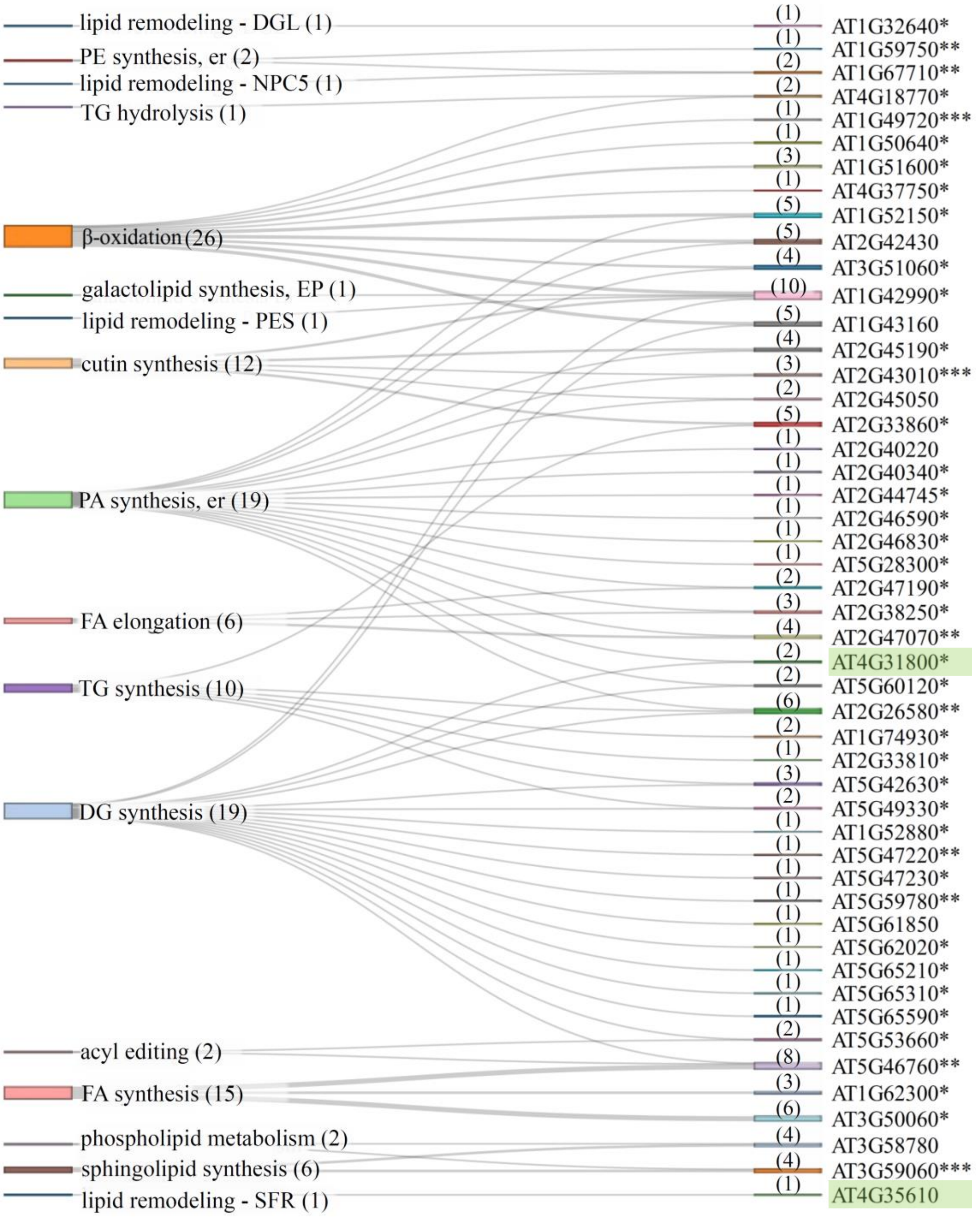

Supplementary Figure 7. Candidate genes identified for Arabidopsis accessions under 6 days of darkness. The diagram includes only the candidates that encode transcription factors (right) that contain DNA-binding domains for the genes of the GPR rules for which the candidate was identified. Traits are grouped by the metabolic pathway to which they belong (left). Source data are provided as a Source Data file.

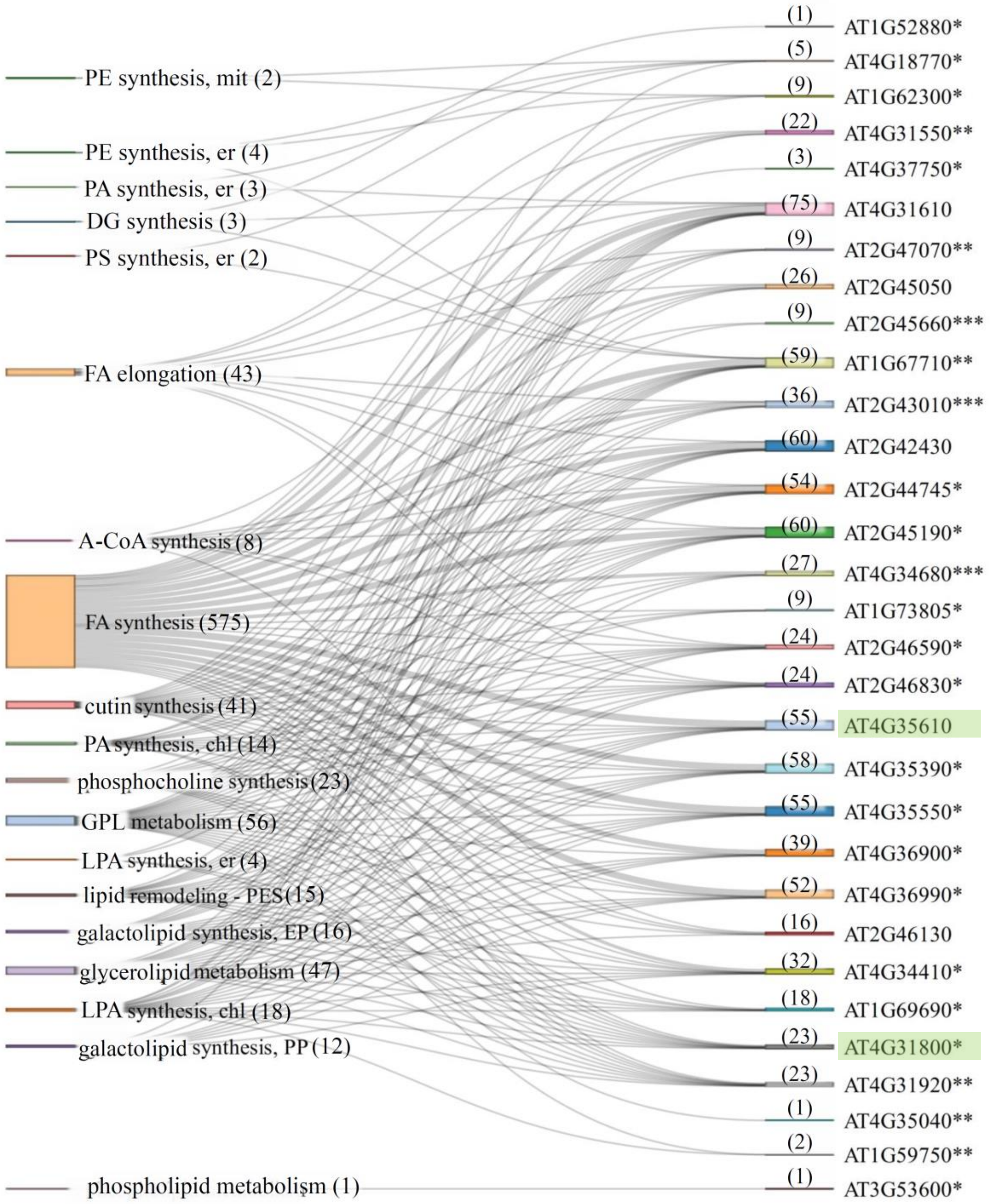

Supplementary Figure 8. Candidate genes identified for control samples of Arabidopsis accessions under 6 days of darkness. The diagram includes only the candidates that encode transcription factors (right) that contain DNA-binding domains for the genes of the GPR rules for which the candidate was identified. Traits are grouped by the metabolic pathway to which they belong (left). Source data are provided as a Source Data file.

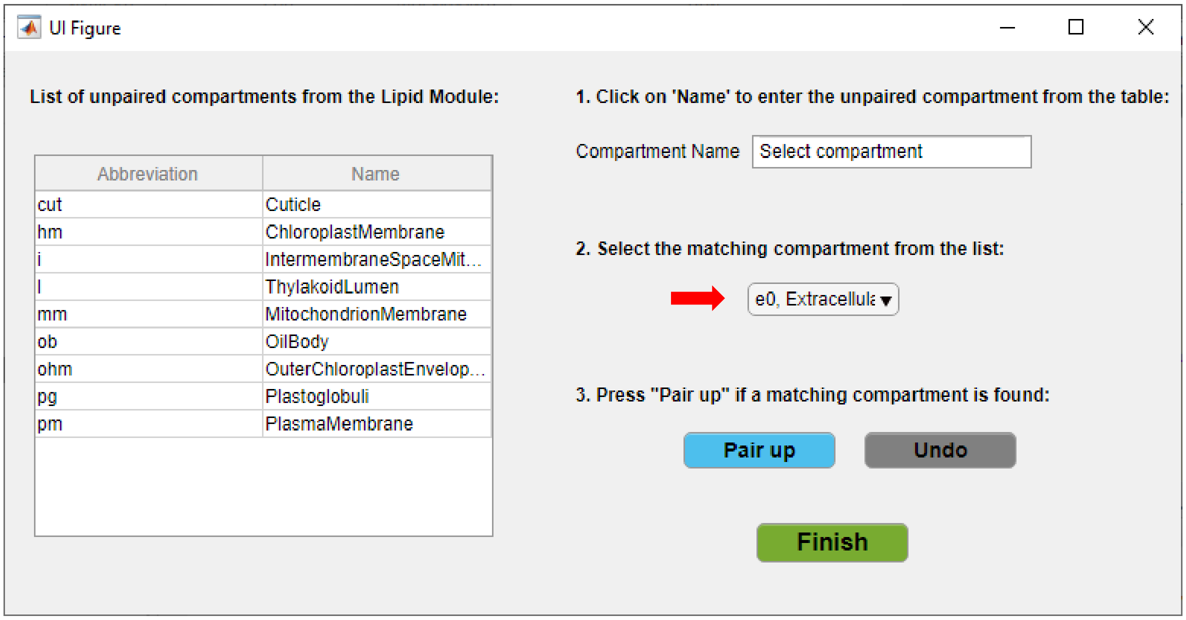

Supplementary Figure 9. User interface of the "LipidModuleIntegration" software tool that allows to manually pair up the compartments of two metabolic models.

Supplementary Table 1. The functionality of the software integration tool was tested with several medium size and genome-scale metabolic (GEM) models. The time (minutes) required for the integration of the PLM was estimated using an ASUS laptop, with 11th Gen Intel Core i5, 3.11 GHz processor, 16.0 RAM and 64 bits operating system.

Model used as template	Features of model				Time required for integration (min.)	Reference
	Size	Reactions	Metabolites	Compartments		
Arabidopsis model (Path2Models project)	GEM	3269	1583	3	~ 1.76	105
AraCore model	Medium	550	406	6	~ 1.01	38
Evidenced-Arabidopsis-Model	GEM	2841	2864	10	~ 1.35	54
CAM diel model	Medium	637	552	13	~ 0.7	106
Jatropha model	Medium	761	633	8	~ 0.6	107

Supplementary Table 2. The synthetic lethality analysis was performed with the AraCore model[38] before and after the integration of the Plant Lipid Module (PLM). The table below includes the number of common and unique genes identified in the single- and double-lethal sets. The complete set of lethal genes obtained for the AraCore model before and after its expansion with the PLM can be found in the Source Data file.

**Lethality analysis**	**Metabolic model**	**Total lethal genes identified**	**Unique genes AraCore**	**Unique genes AraCore expanded**	**Common genes**
SLG	AraCore	108	8 (4.4%)	74 (40.7%)	100 (54.9%)
	AraCore expanded	174			
DLG	AraCore	113	21 (9.9%)	99 (46.7%)	92 (43.4%)
	AraCore expanded	191			

DLG, double lethal gene; SLG, single lethal gene

Supplementary Table 3. **The impact of gene mutation on the synthesis of biomass precursors was estimated by classifying the reactions catalyzed by the respective gene product according to the biomass precursor (or its intermediates) that they produce.** The total number of genes for each metabolite class was calculated and the results were expressed as percentage (%) of missing precursors upon single- and double-gene deletion. Note that the number of lethal genes underlying lipid metabolism is higher than for other metabolite classes because after integrating the Plant Lipid Module into the template model, 76% (4749) of the total reactions (6233) in the model correspond to lipid-related reactions. Details on the classification of the lethal gene sets according to the subsystem and metabolite classes affected by their deletion are included in the Source Data file.

**Metabolite class**	**Single lethal gene (SLG) set**		**Double lethal gene (DLG) set**	
	**Lethal genes per class**	**% missing precursors**	**Lethal genes per class**	**% missing precursors**
Amino acids	33	19.0	32.0	16.8
ATP production	10	5.7	24.0	12.6
Carbohydrates	3	1.7	4.0	2.1
Carbon fixation	24	13.8	8.0	4.2
Cofactors	29	16.7	26.0	13.6
Dicarboxylic acids	1	0.6	0.0	0.0
Lipids	38	21.8	65.0	34.0
Nucleotide sugars	0	0.0	4.0	2.1
Photosynthetic pigments	12	6.9	8.0	4.2
Secondary metabolites	14	8.0	14.0	7.3
Sugar phosphates	2	1.1	6.0	3.1
Transport	8	4.6	0.0	0.0

Supplementary Table 4. List of candidate genes identified for Arabidopsis accessions under control and extended darkness conditions. A genome-wide association study was carried out for Arabidopsis accessions exposed 3 (3DD) and 6 (6DD) days to extended darkness, using reaction fluxes as intermediate traits. The table below summarizes the information about the total number of candidate genes identified for each condition, the number of candidates that were predicted to modulate lipid metabolism, and the number of such candidates that could be validated using mutant lines. The list of significant SNPs identified for each trait and condition evaluated is presented in the Source Data file.

Summary candidate genes identified	3DD	%	Control-3DD	%	6DD	%	Control-6DD	%
Total candidate genes identified[a]	12256		5402		13439		8227	
Enzyme-encoding candidate genes modulating lipid metabolism[b]	11	0.09	9	0.17	16	0.12	24	0.29
Enzyme-encoding candidates validated[c]	2	**18.18**	0	**0.00**	3	**18.75**	1	**4.17**
TF-encoding candidate genes modulating lipid metabolism[b]	33	0.27	12	0.22	49	0.36	31	0.38
TF-encoding candidates validated[d]	14	**42.42**	1	**8.33**	15	**30.61**	21	**67.74**
Total candidates validated	16	36.36	1	4.76	18	27.69	22	40.00

[a] The calculation was made for each condition by counting the candidate genes identified for all the traits evaluated (see column locus in Source Data file).

[b] The candidate genes were classified according to the functional roles fulfilled by the proteins they encoded, for instance, enzymes and transcription factors (TFs). Their role in the modulation of lipid metabolism was predicted by mapping each candidate to the GPR rules of the model, and to information from protein-protein interaction (AthaMap database, http://www.athamap.de/index.php).

[c] Data of lipid profiles measured for T-DNA lines were used to validate the enzyme-encoding candidate genes predicted to modulate lipid metabolism. For more details see the Supplementary Data 14 and 18.

[d] Data of lipid profiles measured for T-DNA lines were used to validate the TF-encoding candidate genes predicted to modulate lipid metabolism. For more details see the Supplementary Data 15.

Supplementary Table 5. Classification of traits according to the functional role of proteins encoded by candidate genes. The candidate genes identified for each trait (reaction flux), were classified according to the functional roles fulfilled by the proteins they encoded, for instance, enzymes and transcription factors (TFs). Enzyme-coding genes were identified by mapping the candidate genes to the GPR rules of the corresponding traits. TFs were identified by using the information of AthaMap database[a]. The summary table of the classification is included below. The complete information about the type of candidate genes identified for each trait in the different environmental conditions evaluated, is included in the Source Data file.

**Classification of traits according to functional role of proteins encoded by candidate genes**	**3DD**	**%**	**Control 3DD**	**%**	**6DD**	**%**	**Control 6DD**	**%**
[b]Total of traits for which candidate genes were obtained	460		635		426		397	
[c]Traits for which type of protein encoded by candidate genes is unknown	170	**36.96**	224	**35.28**	144	**33.80**	84	**21.16**
Traits with unknown candidates and no GPR rules assigned	29	**17.06**	45	**20.09**	15	**10.42**	12	**14.29**
Traits with candidate genes encoding proteins matching gene(s) of underlying GPR rule	3	**0.65**	3	**0.47**	8	**1.88**	20	**5.04**
[a]Traits with candidate genes encoding TFs	161	35.00	127	20.00	172	40.38	277	69.77
Traits with TF-candidates with potential DNA-binding domains for the genes of the underlying GPR rules	96	**59.63**	76	**59.84**	92	**53.49**	179	**64.62**
[d]*Traits with TF-candidates with potential binding domains associated with changes in lipid profiles*	8	8.33	2	2.63	8	8.70	1	0.56
[e]Traits with candidate genes encoding protein of GPR rule upstream	12	**2.61**	20	**3.15**	37	**8.69**	83	**20.91**
[e]Traits with candidate genes encoding protein of GPR rule downstream	12	**2.61**	23	**3.62**	45	**10.56**	101	**25.44**
[f]Traits with enzyme-encoding candidates related to GPR rule	23	5.00	35	5.51	53	12.44	129	32.49
[g]Traits with enzyme-encoding candidates associated with changes in lipid profiles	4	17.39	0	0.00	5	9.43	3	2.33
[h]Traits with candidate genes encoding protein of GPR rules not directly related	285	61.96	406	63.94	273	64.08	290	73.05

DD, days of extended darkness

[a] The transcription factors (TFs) were obtained from AthaMap database (http://www.athamap.de/index.php). This information was used in turn to identify the candidate genes encoding for TFs.

[b] The candidate genes identified for each reaction flux (trait) evaluated is included in the Source Data file.

[c] Unknown candidates refer to candidate genes that did not match any of the enzymatic genes included in the GPR rules of the expanded AraCore model, nor did they match the list of TFs in the AthaMap database. Details about the traits related to transport reactions and for which unknown candidates were identified can be found in the Source Data file.

[d] Details about the traits for which the TF-encoding candidates were related to changes in lipid profiles can be found in the Supplementary Data 15.

[e] The candidate genes were mapped to the genes of the GPR rules of reactions immediately upstream and downstream of the corresponding trait (reaction flux). The goal was to determine if the candidate genes were involved in the production of metabolic precursors consumed by the reaction underlying the trait, or to the consumption of metabolite products generated by the reaction underlying the trait.

[f] The traits with enzyme-encoding candidates were counted taking into account the cases in which the candidates exactly matched the GPR rule associated to the trait, and those that matched GPR rules of reactions immediately upstream or upstream the trait.

[g] Details about the traits for which the enzyme-encoding candidates were related to changes in lipid profiles can be found in the Supplementary Data 14 and 18.

[h] Candidate genes that match the GPR rule of a reaction that is in no way related to the trait.

Supplementary Note 1. Mapping of candidate genes identified by GWAS to transcription factor database information

The information contained in the AthaMap database[146], was used to select the candidate genes encoding Transcription Factors (TFs). The list was further filtered by selecting only TF-encoding candidates for lipid-related traits (reaction fluxes). For each TF-encoding candidate, a list of genes with potential binding sites was obtained[146], which was then mapped to the GPR rules of the underlying reaction (see Methods and Supplementary Table 5). The traits for which TF-encoding candidates containing DNA-binding domains for the genes of the corresponding GPR rules, were subsequently classified according to the metabolic pathway to which they belong.

The results obtained for the Arabidopsis accessions exposed to 3 (3DD) and 6 (6DD) days of extended darkness, with their respective control samples, are summarized in the Supplementary Figs. 5-8. The columns on the right-hand side of the figures contain the list of TFs which are identified by their locus name. Each TF is accompanied by a number that indicates the traits for which the corresponding GPR rule genes contain potential binding sites for said TF.

The asterisks, for their part, refer to the levels of expression of the TFs in whole leaves[3]. The TFs were classified into four categories according to their level of expression: (i) [*] – low, <0.3; (ii) [**] – intermediate, 0.3≤ ≤0.6; and (iii) [***] – high, >0.6. The absence of an asterisk indicates that there is no evidence that the TF is expressed in leaves.

Two TFs, *WRKY18* (AT4G31800) and *TREE1* (AT4G35610) (highlighted in green in Supplementary Figs. 5-6), were identified as candidate genes in fluxes predicted for control (light) and extended darkness conditions. More details about their patterns of expression and their biological role can be found in the Results section of the manuscript.

The TF with locus name AT3G54320 (red font color) (see Supplementary Figs. 5-6), also known as *WRINKLED 1* (*WRI1*), is the target of several master regulators that controls embryo development and maturation[147]. *WRI1*, for its part, controls the expression of several enzymes of the glycolytic, as well as the fatty acid synthesis pathway[75,78,80,148–156]. *WRI1* is expressed not only in seeds, but also in leaves, and therefore its overexpression in the latter tissue has become part of an integral strategy to increase lipid content in vegetative tissues[157 158 159].

Lastly, the left column of Supplementary Figs. 5-8 corresponds to the metabolic pathways of the trait for which the respective TF-encoding candidate was identified.

Supplementary references

1. Berardini, T. Z. *et al.* The Arabidopsis information resource: making and mining the 'gold standard' annotated reference plant genome. *Genesis* **53**, 474–485 (2015).
2. Hooper, C. M., Castleden, I. R., Tanz, S. K., Aryamanesh, N. & Millar, A. H. SUBA4: the interactive data analysis centre for Arabidopsis subcellular protein locations. *Nucleic Acids Res.* **45**, D1064–D1074 (2017).
3. Klepikova, A. V., Kasianov, A. S., Gerasimov, E. S., Logacheva, M. D. & Penin, A. A. A high resolution map of the *Arabidopsis thaliana* developmental transcriptome based on RNA-seq profiling. *Plant J.* **88**, 1058–1070 (2016).
4. Schwacke, R. *et al.* ARAMEMNON, a novel database for Arabidopsis integral membrane proteins. *Plant Physiol.* **131**, 16–26 (2003).
5. Obayashi, T., Aoki, Y., Tadaka, S., Kagaya, Y. & Kinoshita, K. ATTED-II in 2018: a plant coexpression database based on investigation of the statistical property of the mutual rank index. *Plant Cell Physiol.* **59**, E3 (2018).
6. Li, P. *et al.* AtPID: the overall hierarchical functional protein interaction network interface and analytic platform for Arabidopsis. *Nucleic Acids Res.* **39**, D1130–D1133 (2011).
7. Rastogi, S. & Rost, B. LocDB: experimental annotations of localization for Homo sapiens and *Arabidopsis thaliana*. *Nucleic Acids Res.* **39**, D230–D234 (2011).
8. Kanehisa, M. & Goto, S. KEGG: kyoto encyclopedia of genes and genomes. *Nucleic Acids Res.* **28**, 27–30 (2000).
9. The UniProt Consortium *et al.* UniProt: the universal protein knowledgebase in 2021. *Nucleic Acids Res.* **49**, D480–D489 (2021).
10. Schomburg, I. *et al.* BRENDA in 2013: integrated reactions, kinetic data, enzyme function data, improved disease classification: new options and contents in BRENDA. *Nucleic Acids Res.* **41**, D764–D772 (2013).
11. Mueller, L. A., Zhang, P. & Rhee, S. Y. AraCyc: a biochemical pathway database for Arabidopsis. *Plant Physiol.* **132**, 453–460 (2003).

12. Li-Beisson, Y. *et al.* Acyl-lipid metabolism. in *The Arabidopsis book* vol. 8 e0161 (American Society of Plant Biologists, 2010).

13. Lan, Y. *et al.* AtMAD: *Arabidopsis thaliana* multi-omics association database. *Nucleic Acids Res.* **49**, D1445–D1451 (2021).

14. Martens, M. *et al.* WikiPathways: connecting communities. *Nucleic Acids Res.* **49**, D613–D621 (2021).

15. Alonso, A. P., Goffman, F. D., Ohlrogge, J. B. & Shachar-Hill, Y. Carbon conversion efficiency and central metabolic fluxes in developing sunflower (Helianthus annuus L.) embryos. *Plant J.* **52**, 296–308 (2007).

16. Schwender, J., Goffman, F., Ohlrogge, J. B. & Shachar-Hill, Y. Rubisco without the Calvin cycle improves the carbon efficiency of developing green seeds. *Nature* **432**, 779–782 (2004).

17. Kruger, N. J., Masakapalli, S. K. & Ratcliffe, R. G. Strategies for investigating the plant metabolic network with steady-state metabolic flux analysis: lessons from an Arabidopsis cell culture and other systems. *J. Exp. Bot.* **63**, 2309–2323 (2012).

18. Allen, D. K. & Young, J. D. Carbon and nitrogen provisions alter the metabolic flux in developing soybean embryos. *Plant Physiol* **161**, 1458–1475 (2013).

19. Masakapalli, S. K., Kruger, N. J. & Ratcliffe, R. G. The metabolic flux phenotype of heterotrophic Arabidopsis cells reveals a complex response to changes in nitrogen supply. *Plant J.* **74**, 569–582 (2013).

20. Colombié, S. *et al.* Modelling central metabolic fluxes by constraint-based optimization reveals metabolic reprogramming of developing *Solanum lycopersicum* (tomato) fruit. *Plant J.* **81**, 24–39 (2015).

21. Schwender, J. *et al.* Quantitative multilevel analysis of central metabolism in developing oilseeds of oilseed rape during in vitro culture. *Plant Physiol.* **168**, 828–848 (2015).

22. Rossi, M. T., Kalde, M., Srisakvarakul, C., Kruger, N. J. & George Ratcliffe, R. Cell-type specific metabolic flux analysis: a challenge for metabolic phenotyping and a potential solution in plants. *Metabolites* **7**, 59 (2017).

23. Cocuron, J. C., Koubaa, M., Kimmelfield, R., Ross, Z. & Alonso, A. P. A combined metabolomics and fluxomics analysis identifies steps limiting oil synthesis in Maize

embryos. *Plant Physiol.* **181**, 961–975 (2019).

24. Schwender, J., Shachar-Hill, Y. & Ohlrogge, J. B. Mitochondrial metabolism in developing embryos of Brassica napus. *J. Biol. Chem.* **281**, 34040–34047 (2006).

25. Junker, B. H., Lonien, J., Heady, L. E., Rogers, A. & Schwender, J. Parallel determination of enzyme activities and in vivo fluxes in Brassica napus embryos grown on organic or inorganic nitrogen source. *Phytochemistry* **68**, 2232–2242 (2007).

26. Alonso, A. P., Raymond, P., Rolin, D. & Dieuaide-Noubhani, M. Substrate cycles in the central metabolism of maize root tips under hypoxia. *Phytochemistry* **68**, 2222–2231 (2007).

27. Williams, T. C. R. *et al.* Metabolic network fluxes in heterotrophic Arabidopsis cells: stability of the flux distribution under different oxygenation conditions. *Plant Physiol.* **148**, 704–718 (2008).

28. Allen, D. K., Ohlrogge, J. B. & Shachar-Hill, Y. The role of light in soybean seed filling metabolism. *Plant J.* **58**, 220–234 (2009).

29. Lonien, J. & Schwender, J. Analysis of metabolic flux phenotypes for two arabidopsis mutants with severe impairment in seed storage lipid synthesis. *Plant Physiol.* **151**, 1617–1634 (2009).

30. Alonso, A. P., Dale, V. L. & Shachar-Hill, Y. Understanding fatty acid synthesis in developing maize embryos using metabolic flux analysis. *Metab. Eng.* **12**, 488–497 (2010).

31. Alonso, A. P., Val, D. L. & Shachar-Hill, Y. Central metabolic fluxes in the endosperm of developing maize seeds and their implications for metabolic engineering. *Metab. Eng.* **13**, 96–107 (2011).

32. Grafahrend-Belau, E., Schreiber, F., Koschützki, D. & Junker, B. H. Flux balance analysis of barley seeds: a computational approach to study systemic properties of central metabolism. *Plant Physiol.* **149**, 585–598 (2009).

33. Hay, J. & Schwender, J. Computational analysis of storage synthesis in developing Brassica napus L. (oilseed rape) embryos: flux variability analysis in relation to 13C metabolic flux analysis. *Plant J.* **67**, 513–525 (2011).

34. Hay, J. & Schwender, J. Metabolic network reconstruction and flux variability

analysis of storage synthesis in developing oilseed rape (*Brassica napus* L.) embryos. *Plant J.* **67**, 526–541 (2011).

35. Schwender, J. & Hay, J. O. Predictive modeling of biomass component tradeoffs in Brassica napus developing oilseeds based on in silico manipulation of storage metabolism. *Plant Physiol.* **160**, 1218–1236 (2012).

36. Pilalis, E., Chatziioannou, A., Thomasset, B. & Kolisis, F. An in silico compartmentalized metabolic model of *Brassica napus* enables the systemic study of regulatory aspects of plant central metabolism. *Biotechnol. Bioeng.* **108**, 1673–1682 (2011).

37. Grafahrend-Belau, E. *et al.* Multiscale metabolic modeling: dynamic flux balance analysis on a whole-plant scale. *Plant Physiol.* **163**, 637–647 (2013).

38. Arnold, A. & Nikoloski, Z. Bottom-up metabolic reconstruction of Arabidopsis and its application to determining the metabolic costs of enzyme production. *Plant Physiol.* **165**, 1380–1391 (2014).

39. Poolman, M. G., Miguet, L., Sweetlove, L. J. & Fell, D. A. A genome-scale metabolic model of Arabidopsis and some of its properties. *Plant Physiol.* **151**, 1570–1581 (2009).

40. Dal'Molin, C. G. de O., Quek, L. E., Palfreyman, R. W., Brumbley, S. M. & Nielsen, L. K. AraGEM, a genome-scale reconstruction of the primary metabolic network in Arabidopsis. *Plant Physiol.* **152**, 579–589 (2010).

41. Bogart, E. & Myers, C. R. Multiscale metabolic modeling of C4 plants: connecting nonlinear genome-scale models to leaf-scale metabolism in developing maize leaves. *PLoS One* **11**, e0151722 (2016).

42. Monaco, M. K. *et al.* Maize metabolic network construction and transcriptome analysis. *Plant Genome* **6**, 1–12 (2013).

43. Chatterjee, A., Huma, B., Shaw, R. & Kundu, S. Reconstruction of *Oryza sativa* indica genome scale metabolic model and its responses to varying RuBisCO activity, light intensity, and enzymatic cost conditions. *Front. Plant Sci.* **8**, 2060 (2017).

44. Botero, K., Restrepo, S. & Pinzón, A. A genome-scale metabolic model of potato late blight suggests a photosynthesis suppression mechanism. *BMC Genomics* **19**, 31–44 (2018).

45. Pfau, T. *et al.* The intertwined metabolism during symbiotic nitrogen fixation elucidated by metabolic modelling. *Sci. Rep.* **8**, 1–11 (2018).

46. Shaw, R. & Cheung, C. Y. M. A dynamic multi-tissue flux balance model captures carbon and nitrogen metabolism and optimal resource partitioning during Arabidopsis growth. *Front. Plant Sci.* **9**, 884 (2018).

47. Moreira, T. B. *et al.* A genome-scale metabolic model of soybean (*Glycine max*) highlights metabolic fluxes in seedlings. *Plant Physiol.* **180**, 1912–1929 (2019).

48. Radrich, K. *et al.* Integration of metabolic databases for the reconstruction of genome-scale metabolic networks. *Bmc Syst. Biol.* **4**, 114 (2010).

49. Saha, R., Suthers, P. F. & Maranas, C. D. Zea mays irs1563: a comprehensive genome-scale metabolic reconstruction of maize metabolism. *PLoS One* **6**, e21784 (2011).

50. Mintz-Oron, S. *et al.* Reconstruction of Arabidopsis metabolic network models accounting for subcellular compartmentalization and tissue-specificity. *Proc. Natl. Acad. Sci. U. S. A.* **109**, 339–344 (2012).

51. Poolman, M. G., Kundu, S., Shaw, R. & Fell, D. A. Responses to light intensity in a genome-scale model of rice metabolism. *Plant Physiol.* **162**, 1060–1072 (2013).

52. Simons, M. *et al.* Assessing the metabolic impact of nitrogen availability using a compartmentalized maize leaf genome-scale model. *Plant Physiol.* **166**, 1659–1674 (2014).

53. Lakshmanan, M. *et al.* Unraveling the light-specific metabolic and regulatory signatures of rice through combined in silico modeling and multiomics analysis. *Plant Physiol.* **169**, 3002–3020 (2015).

54. Seaver, S. M. D. *et al.* Improved evidence-based genome-scale metabolic models for maize leaf, embryo, and endosperm. *Front. Plant Sci.* **6**, 142 (2015).

55. Yuan, H., Cheung, M. C. Y., Poolman, M. G., Hilbers, P. A. J. & van Riel, N. A. W. A genome-scale metabolic network reconstruction of tomato (*Solanum lycopersicum* L.) and its application to photorespiratory metabolism. *Plant J.* **85**, 289–304 (2016).

56. Krishnakumar, V. *et al.* Araport: the Arabidopsis information portal. *Nucleic Acids Res.* **43**, D1003–D1009 (2015).

57. Awai, K., Xu, C., Tamot, B. & Benning, C. A phosphatidic acid-binding protein of

the chloroplast inner envelope membrane involved in lipid trafficking. *Proc. Natl. Acad. Sci. U. S. A.* **103**, 10817–10822 (2006).

58. Wang, Z., Xu, C. & Benning, C. TGD4 involved in endoplasmic reticulum-to-chloroplast lipid trafficking is a phosphatidic acid binding protein. *Plant J.* **70**, 614–623 (2012).
59. Hurlock, A. K., Roston, R. L., Wang, K. & Benning, C. Lipid trafficking in plant cells. *Traffic* **15**, 915–932 (2014).
60. Lu, B. & Benning, C. A 25-amino acid sequence of the Arabidopsis TGD2 protein is sufficient for specific binding of phosphatidic acid. *J. Biol. Chem.* **284**, 17420–17427 (2009).
61. Michaud, M. & Jouhet, J. Lipid trafficking at membrane contact sites during plant development and stress response. *Front. Plant Sci.* **10**, 1–10 (2019).
62. Maréchal, E. & Bastien, O. Modeling of regulatory loops controlling galactolipid biosynthesis in the inner envelope membrane of chloroplasts. *J. Theor. Biol.* **361**, 1–13 (2014).
63. Mueller-Schuessele, S. J. & Michaud, M. Plastid transient and stable interactions with other cell compartments. in *Methods in Molecular Biology* (ed. Walker, J. M.) vol. 1829 87–109 (Humana Press Inc., Totowa, NJ, 2018).
64. Andersson, M. X. & Dörmann, P. Chloroplast membrane lipid biosynthesis and transport. in *The Chloroplast. Plant Cell Monographs* (eds. Sandelius, A. S. & Aronsson, H.) 125–158 (Springer-Verlag, Berlin Heidelberg, 2009).
65. LaBrant, E., Barnes, A. C. & Roston, R. L. Lipid transport required to make lipids of photosynthetic membranes. *Photosynth. Res.* **138**, 345–360 (2018).
66. Hamilton, J. A., Bhamidipati, S. P., Kodali, D. R. & Small, D. M. The interfacial conformation and transbilayer movement of diacylglycerols in phospholipid bilayers. *J. Biol. Chem.* **266**, 1177–1186 (1991).
67. Dorne, A. J., Joyard, J., Block, M. A. & Douce, R. Localization of phosphatidylcholine in outer envelope membrane of spinach chloroplasts. *J. Cell Biol.* **100**, 1690–1697 (1985).
68. Bessoule, J. J., Testet, E. & Cassagne, C. Synthesis of phosphatidylcholine in the chloroplast envelope after import of lysophosphatidylcholine from endoplasmic

reticulum membranes. *Eur. J. Biochem.* **228**, 490–497 (1995).

69. Xu, C., Fan, J., Cornish, A. J. & Benning, C. Lipid trafficking between the endoplasmic reticulum and the plastid in Arabidopsis requires the extraplastidic TGD4 protein. *Plant Cell* **20**, 2190–2204 (2008).
70. Li-Beisson, Y., Neunzig, J., Lee, Y. & Philippar, K. Plant membrane-protein mediated intracellular traffic of fatty acids and acyl lipids. *Curr. Opin. Plant Biol.* **40**, 138–146 (2017).
71. Botella, C. *et al.* ALA10, a phospholipid flippase, controls FAD2/FAD3 desaturation of phosphatidylcholine in the ER and affects chloroplast lipid composition in *Arabidopsis thaliana*. *Plant Physiol.* **170**, 1300–1314 (2016).
72. Dubots, E. *et al.* Activation of the chloroplast monogalactosyldiacylglycerol synthase MGD1 by phosphatidic acid and phosphatidylglycerol. *J. Biol. Chem.* **285**, 6003–6011 (2010).
73. Nakamura, Y., Tsuchiya, M. & Ohta, H. Plastidic phosphatidic acid phosphatases identified in a distinct subfamily of lipid phosphate phosphatases with prokaryotic origin. *J. Biol. Chem.* **282**, 29013–29021 (2007).
74. Browse, J. Towards rational engineering of plant oils: crystal structure of the 18:0-ACP desaturase. *Trends Plant Sci.* **1**, 403–404 (1996).
75. Baud, S. & Lepiniec, L. Physiological and developmental regulation of seed oil production. *Prog. Lipid Res.* **49**, 235–249 (2010).
76. Taylor, D. C., Smith, M. A., Fobert, P., Mietkiewska, E. & Weselake, R. J. Metabolic engineering of higher plants to produce bio-industrial oils. in *Comprehensive Biotechnology (Second Edition)* (ed. Moo-Young, M.) 67–85 (Academic Press, Cambridge, MA, 2011).
77. Bates, P. D. Understanding the control of acyl flux through the lipid metabolic network of plant oil biosynthesis. *Biochim. Biophys. Acta - Mol. Cell Biol. Lipids* **1861**, 1214–1225 (2016).
78. Schmid, K. M. Chapter 4 - Lipid metabolism in plants. in *Biochemistry of Lipids, Lipoproteins and Membranes (Sixth Edition)* (eds. Ridgway, N. D. & McLeod, R. S.) 113–147 (Elsevier, Boston, 2016).
79. Singer, S. D., Zou, J. T. & Weselake, R. J. Abiotic factors influence plant storage

lipid accumulation and composition. *Plant Sci.* **243**, 1–9 (2016).

80. Bates, P. D., Stymne, S. & Ohlrogge, J. Biochemical pathways in seed oil synthesis. *Curr. Opin. Plant Biol.* **16**, 358–364 (2013).
81. Allen, D. K., Bates, P. D. & Tjellström, H. Tracking the metabolic pulse of plant lipid production with isotopic labeling and flux analyses: past, present and future. *Prog. Lipid Res.* **58**, 97–120 (2015).
82. Rennie, E. A. *et al.* Identification of a sphingolipid α-glucuronosyltransferase that is essential for pollen function in Arabidopsis. *Plant Cell* **26**, 3314–3325 (2014).
83. Buré, C., Cacas, J.-L., Mongrand, S. & Schmitter, J.-M. Characterization of glycosyl inositol phosphoryl ceramides from plants and fungi by mass spectrometry. *Anal. Bioanal. Chem.* **406**, 995–1010 (2013).
84. Cacas, J. L. *et al.* Biochemical survey of the polar head of plant glycosylinositolphosphoceramides unravels broad diversity. *Phytochemistry* **96**, 191–200 (2013).
85. Tartaglio, V. *et al.* Glycosylation of inositol phosphorylceramide sphingolipids is required for normal growth and reproduction in Arabidopsis. *Plant J.* **89**, 278–290 (2017).
86. Seo, H. S. *et al.* Jasmonic acid carboxyl methyltransferase: a key enzyme for jasmonate-regulated plant responses. *Proc. Natl. Acad. Sci.* **98**, 4788–4793 (2001).
87. Block, M. A. & Jouhet, J. Lipid trafficking at endoplasmic reticulum-chloroplast membrane contact sites. *Curr. Opin. Cell Biol.* **35**, 21–29 (2015).
88. Andersson, M. X., Goksör, M. & Sandelius, A. S. Membrane contact sites. *Plant Signal. Behav.* **2**, 185–187 (2007).
89. Ytterberg, A. J., Peltier, J.-B. & van Wijk, K. J. Protein profiling of plastoglobules in chloroplasts and chromoplasts. A surprising site for differential accumulation of metabolic enzymes. *Plant Physiol.* **140**, 984–997 (2006).
90. Lundquist, P. K. *et al.* The functional network of the Arabidopsis plastoglobule proteome based on quantitative proteomics and genome-wide coexpression analysis. *Plant Physiol.* **158**, 1172–1192 (2012).
91. Moellering, E. R., Muthan, B. & Benning, C. Freezing tolerance in plants requires lipid remodeling at the outer chloroplast membrane. *Science* **330**, 226–228 (2010).

92. Roston, R. L., Wang, K., Kuhn, L. A. & Benning, C. Structural determinants allowing transferase activity in SENSITIVE TO FREEZING 2, classified as a family I glycosyl hydrolase. *J. Biol. Chem.* **289**, 26089–26106 (2014).
93. Li, W. *et al.* Differential degradation of extraplastidic and plastidic lipids during freezing and post-freezing recovery in *Arabidopsis thaliana*. *J. Biol. Chem.* **283**, 461–468 (2008).
94. Higashi, Y. *et al.* HEAT INDUCIBLE LIPASE1 remodels chloroplastic monogalactosyldiacylglycerol by liberating α-linolenic acid in Arabidopsis leaves under heat stress. *Plant Cell* **30**, 1887–1905 (2018).
95. Lippold, F. *et al.* Fatty acid phytyl ester synthesis in chloroplasts of Arabidopsis. *Plant Cell* **24**, 2001–2014 (2012).
96. Higashi, Y., Okazaki, Y., Myouga, F., Shinozaki, K. & Saito, K. Landscape of the lipidome and transcriptome under heat stress in *Arabidopsis thaliana*. *Sci. Rep.* **5**, 1–11 (2015).
97. Gaude, N., Nakamura, Y., Scheible, W.-R., Ohta, H. & Dörmann, P. Phospholipase C5 (NPC5) is involved in galactolipid accumulation during phosphate limitation in leaves of Arabidopsis. *Plant J.* **56**, 28–39 (2008).
98. Nakamura, Y. Phosphate starvation and membrane lipid remodeling in seed plants. *Prog. Lipid Res.* **52**, 43–50 (2013).
99. Jouhet, J. *et al.* Phosphate deprivation induces transfer of DGDG galactolipid from chloroplast to mitochondria. *J. Cell Biol.* **167**, 863–874 (2004).
100. Michaud, M. *et al.* AtMic60 is involved in plant mitochondria lipid trafficking and is part of a large complex. *Curr. Biol.* **26**, 627–639 (2016).
101. Sanchez, B. J., Li, F., Kerkhoven, E. J. & Nielsen, J. SLIMEr: probing flexibility of lipid metabolism in yeast with an improved constraint-based modeling framework. *Bmc Syst. Biol.* **13**, 4 (2019).
102. Ponce-de-León, M., Montero, F. & Peretó, J. Solving gap metabolites and blocked reactions in genome-scale models: application to the metabolic network of *Blattabacterium cuenoti*. *BMC Syst. Biol.* **7**, 114 (2013).
103. Thiele, I. & Palsson, B. A protocol for generating a high-quality genome-scale metabolic reconstruction. *Nat. Protoc.* **5**, 93–121 (2010).

104. Liebisch, G. *et al.* Update on LIPID MAPS classification, nomenclature, and shorthand notation for MS-derived lipid structures. *J. Lipid Res.* **61**, 1539–1555 (2020).

105. Büchel, F. *et al.* Path2Models: large-scale generation of computational models from biochemical pathway maps. *BMC Syst. Biol.* **7**, 1–19 (2013).

106. Shameer, S., Baghalian, K., Cheung, C. Y. M., Ratcliffe, R. G. & Sweetlove, L. J. Computational analysis of the productivity potential of CAM. *Nat. Plants* **4**, 165–171 (2018).

107. Correa, S. M. *et al.* Model-assisted identification of metabolic engineering strategies for *Jatropha curcas* lipid pathways. *Plant J.* **104**, 76–95 (2020).

108. Schellenberger, J. *et al.* Quantitative prediction of cellular metabolism with constraint-based models: the COBRA toolbox v2.0. *Nat. Protoc.* **6**, 1290–1307 (2011).

109. Heirendt, L. *et al.* Creation and analysis of biochemical constraint-based models using the COBRA toolbox v.3.0. *Nat. Protoc.* **14**, 639–702 (2019).

110. Correa, S. M. et al. Identification of gene function based on models capturing natural variability of *Arabidopsis thaliana* lipid metabolism. marce2336/PlantLipidModule. DOI:10.5281/zenodo.8179057 (2023).

111. Wang, M., Herrmann, C., Simonovic, M., Szklarczyk, D. & von Mering, C. Version 4.0 of PaxDb: protein abundance data, integrated across model organisms, tissues, and cell-lines. *Proteomics* **15**, 3163–3168 (2015).

112. Gfeller, A. *et al.* Jasmonate controls polypeptide patterning in undamaged tissue in wounded Arabidopsis leaves. *Plant Physiol.* **156**, 1797–1807 (2011).

113. Mergner, J. *et al.* Proteomic and transcriptomic profiling of aerial organ development in Arabidopsis. *Sci. Data* **7**, 334 (2020).

114. Wang, J. *et al.* Proteomic insight into the response of Arabidopsis chloroplasts to darkness. *PLoS One* **11**, e0154235 (2016).

115. Gibon, Y. *et al.* Adjustment of growth, starch turnover, protein content and central metabolism to a decrease of the carbon supply when Arabidopsis is grown in very short photoperiods. *Plant. Cell Environ.* **32**, 859–874 (2009).

116. Tschoep, H. *et al.* Adjustment of growth and central metabolism to a mild but

sustained nitrogen-limitation in Arabidopsis. *Plant. Cell Environ.* **32**, 300–318 (2009).

117. Sulpice, R. *et al.* Impact of the carbon and nitrogen supply on relationships and connectivity between metabolism and biomass in a broad panel of Arabidopsis accessions. *Plant Physiol.* **162**, 347–363 (2013).

118. Horton, M. W. *et al.* Genome-wide patterns of genetic variation in worldwide *Arabidopsis thaliana* accessions from the RegMap panel. *Nat. Genet.* **44**, 212–216 (2012).

119. Baxter, I. *et al.* A coastal cline in sodium accumulation in *Arabidopsis thaliana* is driven by natural variation of the sodium transporter AtHKT1;1. *PLOS Genet.* **6**, e1001193 (2010).

120. DeBolt, S. *et al.* Mutations in UDP-glucose:sterol glucosyltransferase in Arabidopsis cause transparent testa phenotype and suberization defect in seeds. *Plant Physiol.* **151**, 78–87 (2009).

121. Smith, A. M. & Zeeman, S. C. Quantification of starch in plant tissues. *Nat. Protoc.* **1**, 1342–1345 (2006).

122. Sharrock, R. A. & Clack, T. Patterns of expression and normalized levels of the five Arabidopsis phytochromes. *Plant Physiol.* **130**, 442–456 (2002).

123. Dörmann, P., Hoffmann-Benning, S., Balbo, I. & Benning, C. Isolation and characterization of an Arabidopsis mutant deficient in the thylakoid lipid digalactosyl diacylglycerol. *Plant Cell* **7**, 1801–1810 (1995).

124. Liu, N. J. *et al.* Lipidomic analysis reveals the importance of GIPCs in Arabidopsis leaf extracellular vesicles. *Mol. Plant* **13**, 1523–1532 (2020).

125. Lü, S. *et al.* Arabidopsis CER8 encodes LONG-CHAIN ACYL-COA SYNTHETASE 1 (LACS1) that has overlapping functions with LACS2 in plant wax and cutin synthesis. *Plant J.* **59**, 553–564 (2009).

126. Weraduwage, S. M. *et al.* The relationship between leaf area growth and biomass accumulation in *Arabidopsis thaliana*. *Front. Plant Sci.* **6**, 1–21 (2015).

127. Li, Y. *et al.* Identification of acyltransferases required for cutin biosynthesis and production of cutin with suberin-like monomers. *Proc. Natl. Acad. Sci. U. S. A.* **104**, 18339–18344 (2007).

128. Araújo, W. L. *et al.* Identification of the 2-hydroxyglutarate and isovaleryl-CoA dehydrogenases as alternative electron donors linking lysine catabolism to the electron transport chain of Arabidopsis mitochondria. *Plant Cell* **22**, 1549–1563 (2010).

129. Hummel, J. *et al.* Ultra performance liquid chromatography and high resolution mass spectrometry for the analysis of plant lipids. *Front. Plant Sci.* **12**, 1–17 (2011).

130. Pratapa, A., Balachandran, S. & Raman, K. Fast-SL: an efficient algorithm to identify synthetic lethal sets in metabolic networks. *Bioinformatics* **31**, 3299–3305 (2015).

131. Szecowka, M. *et al.* Metabolic fluxes in an illuminated Arabidopsis rosette. *Plant Cell* **25**, 694–714 (2013).

132. Meinke, D. W. Genome-wide identification of EMBRYO-DEFECTIVE (EMB) genes required for growth and development in Arabidopsis. *New Phytol.* **226**, 306–325 (2020).

133. Cheung, C. Y., Poolman, M. G., Fell, D. A., George Ratcliffe, R. & Sweetlove, L. J. A diel flux balance model captures interactions between light and dark metabolism during day-night cycles in C3 and Crassulacean acid metabolism leaves. *Plant Physiol.* **165**, 917–929 (2014).

134. Neuhaus, H. E. Transport of primary metabolites across the plant vacuolar membrane. *FEBS Lett.* **581**, 2223–2226 (2007).

135. Huang, X. Y., Wang, C. K., Zhao, Y. W., Sun, C. H. & Hu, D. G. Mechanisms and regulation of organic acid accumulation in plant vacuoles. *Hortic. Res.* **8**, 1–10 (2021).

136. Hurth, M. A. *et al.* Impaired pH homeostasis in Arabidopsis lacking the vacuolar dicarboxylate transporter and analysis of carboxylic acid transport across the tonoplast. *Plant Physiol.* **137**, 901–910 (2005).

137. Yang, G., Wei, Q., Huang, H. & Xia, J. Amino acid transporters in plant cells: a brief review. *Plants (Basel)* **9**, 967 (2020).

138. Yu, L., Fan, J., Yan, C. & Xu, C. Starch deficiency enhances lipid biosynthesis and turnover in leaves. *Plant Physiol.* **178**, 118–129 (2018).

139. Ohlrogge, J. B. & Jaworski, J. G. Regulation of fatty acid synthesis. *Annu. Rev.*

Plant Physiol. Plant Mol. Biol. **48**, 109–136 (1997).

140. Bao, X., Focke, M., Pollard, M. & Ohlrogge, J. Understanding in vivo carbon precursor supply for fatty acid synthesis in leaf tissue. *Plant J.* **22**, 39–50 (2000).

141. Caldana, C. *et al.* High-density kinetic analysis of the metabolomic and transcriptomic response of Arabidopsis to eight environmental conditions. *Plant J.* **67**, 869–884 (2011).

142. Usadel, B. *et al.* Global transcript levels respond to small changes of the carbon status during progressive exhaustion of carbohydrates in Arabidopsis rosettes. *Plant Physiol.* **146**, 1834–1861 (2008).

143. Robaina Estevez, S. Context-specific metabolic predictions. (Universität Potsdam, 2017).

144. Yin, L. *et al.* rMVP: a memory-efficient, visualization-enhanced, and parallel-accelerated tool for genome-wide association study. *Genomics. Proteomics Bioinformatics* **19**, 619–628 (2021).

145. Arouisse, B., Korte, A., van Eeuwijk, F. & Kruijer, W. Imputation of 3 million SNPs in the Arabidopsis regional mapping population. *Plant J.* **102**, 872–882 (2020).

146. Hehl, R. & Bülow, L. AthaMap web tools for the analysis of transcriptional and posttranscriptional regulation of gene expression in *Arabidopsis thaliana*. *Methods Mol. Biol.* **1158**, 139–156 (2014).

147. Correa, S. M., Fernie, A. R., Nikoloski, Z. & Brotman, Y. Towards model-driven characterization and manipulation of plant lipid metabolism. *Prog. Lipid Res.* **80**, 101051 (2020).

148. Baud, S. *et al.* WRINKLED1 specifies the regulatory action of LEAFY COTYLEDON2 towards fatty acid metabolism during seed maturation in Arabidopsis. *Plant J.* **50**, 825–838 (2007).

149. Maeo, K. *et al.* An AP2-type transcription factor, WRINKLED1, of *Arabidopsis thaliana* binds to the AW-box sequence conserved among proximal upstream regions of genes involved in fatty acid synthesis. *Plant J.* **60**, 476–487 (2009).

150. Ruuska, S. A., Girke, T., Benning, C. & Ohlrogge, J. B. Contrapuntal networks of gene expression during Arabidopsis seed filling. *Plant Cell* **14**, 1191–1206 (2002).

151. Cernac, A., Andre, C., Hoffmann-Benning, S. & Benning, C. WRI1 is required for

seed germination and seedling establishment. *Plant Physiol.* **141**, 745–757 (2006).

152. Adhikari, N. D., Bates, P. D. & Browse, J. WRINKLED1 rescues feedback inhibition of fatty acid synthesis in hydroxylase-expressing seeds. *Plant Physiol.* **171**, 179–191 (2016).

153. Harwood, J. L. & Guschina, I. A. Regulation of lipid synthesis in oil crops. *FEBS Lett.* **587**, 2079–2081 (2013).

154. Simpson, J. P., Thrower, N. & Ohlrogge, J. B. How did nature engineer the highest surface lipid accumulation among plants? Exceptional expression of acyl-lipid-associated genes for the assembly of extracellular triacylglycerol by Bayberry (*Myrica pensylvanica*) fruits. *Biochim. Biophys. Acta-Molecular Cell Biol. Lipids* **1861**, 1243–1252 (2016).

155. Zhai, Z. Y. *et al.* Trehalose 6-phosphate positively regulates fatty acid synthesis by stabilizing WRINKLED1. *Plant Cell* **30**, 2616–2627 (2018).

156. Fei, W. *et al.* Research advances of WRINKLED1 (WRI1) in plants. *Funct. Plant Biol.* **47**, 185–194 (2020).

157. Sanjaya, Durrett, T. P., Weise, S. E. & Benning, C. Increasing the energy density of vegetative tissues by diverting carbon from starch to oil biosynthesis in transgenic Arabidopsis. *Plant Biotechnol. J.* **9**, 874–883 (2011).

158. Kelly, A. A. *et al.* The SUGAR-DEPENDENT1 lipase limits triacylglycerol accumulation in vegetative tissues of Arabidopsis. *Plant Physiol.* **162**, 1282–1289 (2013).

159. Alameldin, H. *et al.* Production of seed-like storage lipids and increase in oil bodies in corn (Maize; *Zea mays* L.) vegetative biomass. *Ind. Crops Prod.* **108**, 526–534 (2017).
