# Supplementary material for: Identification of gene function based on models capturing natural variability of Arabidopsis thaliana lipid metabolism
Source: Nat Commun. 2023 Aug 14;14:4897. doi: 10.1038/s41467-023-40644-9 (PMC10425450; doi:10.1038/s41467-023-40644-9)
Supplement: Supplementary file 3 — Description of Additional Supplementary Files [file 41467_2023_40644_MOESM3_ESM.pdf]

Description of Additional Supplementary Files

Supplementary Data 1:

"The lipid classes that were included in the reconstruction of the Plant Lipid Module are listed with their respective class abbreviations and related information according to LIPID MAPS nomenclature."

Supplementary Data 2:

"List of the genes of the AraCore model and the Plant Lipid Module, for which the SeedGenes Project database has information for mutants with the corresponding description of their phenotype. The table includes two additional columns with the list of genes that are part of the AraCore model (second column) and the AraCore model expanded with the Plant Lipid Module (third column). For each gene, its classification as single lethal (SLG), double lethal (DLG) and Non-lethal gene is indicated, according to the results of the synthetic lethality analysis. Entries in light gray (NA) correspond to genes that are not present in the respective model. The entries highlighted in blue correspond to genes for which the classification in the lethality analysis does not correspond to the phenotypic description, while the entries highlighted in red are the cases in which the gene classification agrees with the phenotypic description of the mutant."

Supplementary Data 3:

"List of the genes of the AraCore model and the Plant Lipid Module, for which the ARALIPID website has information on the mutants and the corresponding description of their phenotype. The table includes two additional columns with the list of genes that are part of the AraCore model (second column) and the AraCore model expanded with the Plant Lipid Module (third column). For each gene, its classification as single lethal (SLG), double lethal (DLG) and Non-lethal gene is indicated, according to the results of the synthetic lethality analysis. Entries in light gray (NA) correspond to genes that are not present in the respective model. The entries highlighted in blue correspond to genes for which the classification in the lethality analysis does not correspond to the phenotypic description, while the entries highlighted in red are the cases in which the gene classification agrees with the phenotypic description of the mutant."

Supplementary Data 4:

"Number of accessions with active reactions per metabolic pathway. The number of accessions that have active reactions was calculated for each condition (light and dark), and the results were classified per metabolic subsystem. The accession was considered to have active reactions when at least one reaction of the respective subsystem was carrying a non-zero flux."

Supplementary Data 5:

"Lethality background calculated for the set of single lethal genes of Arabidopsis accessions under extended darkness. The set of single lethal genes was obtained for each Arabidopsis accession by implementing the fast-SL algorithm. The proportion of accessions for which the gene was identified as lethal was also determined. The data used for the simulations corresponded to Arabidopsis accessions exposed to 3 days of extended darkness."

Supplementary Data 6:

“Lethality background calculated for the set of double lethal genes of Arabidopsis accessions under extended darkness. The set of double lethal genes was obtained for each Arabidopsis accession by implementing the fast-SL algorithm. The proportion of accessions for which the gene was identified as lethal was also determined. The data used for the simulations corresponded to Arabidopsis accessions exposed to 3 days of extended darkness.”

Supplementary Data 7:

“Transcript levels measured for Arabidopsis plants under extended darkness. The expression profiles of genes were measured for Arabidopsis plants ecotype Col-0 exposed to periods of 21 and 48 h of extended darkness. The list includes the information about the name of the reaction in the model that is catalyzed by the corresponding gene product, the gene product name, and the pathway to which each reaction belongs. Genes were assigned values of -1, 0, or +1, depending upon whether their transcription decreased (pink), remained unaltered (grey), or increased (blue), respectively, compared to plants under standard condition (light).”

Supplementary Data 8:

“List of Post-translational modifications (PTMs) for enzymes with sign change agreement. The enzymes classified as “concordant” corresponded to those in which the direction of changes between fluxes and expression levels agreed when comparing the log2-fold change for flux rates (V) and transcripts abundance (tr) of Arabidopsis plants under control and extended darkness conditions. PTMs data for the concordant enzymes was retrieved from the FAT-PTM database (DOI: 10.1111/tpj.14372, 2019).”

Supplementary Data 9:

“List of Post-translational modifications (PTMs) for outlier enzymes. The enzymes classified as “outliers” corresponded to those in which the direction of changes between fluxes and expression levels disagreed when comparing the log2-fold change for flux rates (V) and transcripts abundance (tr) of Arabidopsis plants under control and extended darkness conditions. PTMs data for the outlier enzymes was retrieved from the FAT-PTM database (DOI: 10.1111/tpj.14372, 2019).”

Supplementary Data 10:

“Classification of enzymes according to their Post-translational modifications (PTMs). The enzymes classified as 'outlier' and 'concordant' were grouped according to the type(s) of PTMs reported in the FAT-PTM database (DOI: 10.1111/tpj.14372, 2019). The PTMs displayed for each case are marked with an 'x'. Enzymes designated as 'concordant' refer to those for which the sign change agreed when comparing the log2-fold change for flux rates (V) and transcripts abundance (tr) of Arabidopsis plants under control and extended darkness conditions. The 'outlier' enzymes refer to the opposite case.”

Supplementary Data 11:

“Details of candidate genes encoding transcription factors that were found associated with the largest number of fluxes.”

Supplementary Data 12:

“Lipid profiles measured in a collection of 364 *Arabidopsis thaliana* T-DNA insertion lines. The lipid profiles were obtained from the supplementary material 'pcp-2022-e-00127-File012' published

by Lusk et al., (2022) (doi:https://doi.org/10.1093/pcp/pcac088). The information about each T-DNA line is included in the Supplementary Data 13. The original data was filtered to select the lipid profiles of untreated (unwounded) leaves, and the lipid species that are included in the AraCore model expanded with the Plant Lipid Module. The wild-type control samples (WT-Col-0), for each line correspond to those identified with the same day number."

Supplementary Data 13:

"Description of *Arabidopsis thaliana* lines with T-DNA insertions. The Salk lines and their associated gene information was obtained from the supplementary material 'pcp-2022-e-00127-File011' published by Lusk et al. (2022) (doi:https://doi.org/10.1093/pcp/pcac088). For more information about the source of the Arabidopsis lines with T-DNA insertions, and how they were handled the reader is referred to the aforementioned publication."

Supplementary Data 14:

"Identification of changes in lipid profiles of Arabidopsis T-DNA lines corresponding to enzyme-coding candidate genes identified by genome-wide association (GWA). Enzyme-coding candidate genes were identified by means of a GWA study in Arabidopsis accessions under control (light) and extended darkness for 3 (3DD) and 6 (6DD) days. The lipid profiles of the T-DNA lines (see Supplementary Data 12) corresponding to candidate genes were used to determine the occurrence of changes in the relative abundance of lipids participating in the reactions associated with each reaction flux ('trait'). In the fourth column, it is indicated if the candidate gene coincides with a gene of the GPR rule associated with the 'trait', or if it coincides with a gene of the GPR rule of a reaction that produces precursors ('upstream') for 'trait' or consumes the products generated by 'trait' ('downstream'). The fifth column indicates the name of the reaction for which the candidate gene matches the corresponding GPR rule. When changes in the relative abundance of lipids were identified, the corresponding average fold-change (FC) was included."

Supplementary Data 15:

"Identification of changes in lipid profiles of Arabidopsis T-DNA lines corresponding to transcription factor (TF)-coding candidate genes identified by genome-wide association (GWA). TF-coding candidate genes were identified by means of a GWA study in Arabidopsis accessions under control (light) and extended darkness for 3 (3DD) and 6 (6DD) days. Since none of the available T-DNA lines (see Supplementary Data 12) included modification of TF-coding genes, we searched for an indirect association between the TF-coding candidates and changes in the lipid profiles. For this, we selected the genes associated to the GPR rule of the reaction flux ('trait'), that contained potential DNA-binding domains (third column) for the TF-candidates (fourth column). The occurrence of changes in lipid abundance was then determined for the lipids participating in the reactions associated to the trait. When changes in the relative abundance of lipids were identified, the corresponding average fold-change (FC) was included."

Supplementary Data 16:

"List of transport reactions included in the reconstruction of the Plant Lipid Module. Information concerning the mechanism, the justification for the inclusion of each reaction, and the respective literature references available for each case are reported here."

Supplementary Data 17:

“The FVA flux distribution was obtained at optimum biomass (constraining the biomass reaction with the maximum-predicted growth rate) and minimizing flux through gene associated reactions by holding minimum network flux constant. The latter was done by first minimizing the absolute value of flux through all gene-associated reactions via pFBA, and next using this flux to constrain the upper bound for the summed network flux.”

Supplementary Data 18:

“Comparison of flux rates of reactions associated to candidate genes validated with lipidomics data. To further examine the candidate genes validated with lipidomics data (see Supplementary Data 14 and 15), Arabidopsis accessions were classified according to the type of SNPs present in each candidate. The absolute values of the fluxes of the reactions associated with each group of accessions were compared to determine to what extent reaction rates may be influenced by the allele carried by the accessions.”
