# Supplementary material for: Identification of gene function based on models capturing natural variability of Arabidopsis thaliana lipid metabolism
Source: Nat Commun. 2023 Aug 14;14:4897. doi: 10.1038/s41467-023-40644-9 (PMC10425450; doi:10.1038/s41467-023-40644-9)
Supplement: Supplementary file 22 — Reporting Summary [file 41467_2023_40644_MOESM22_ESM.pdf]

nature portfolio

Corresponding author(s): Zoran Nikoloski

Last updated by author(s): Mar 27, 2023

Reporting Summary

Nature Portfolio wishes to improve the reproducibility of the work that we publish. This form provides structure for consistency and transparency in reporting. For further information on Nature Portfolio policies, see our Editorial Policies and the Editorial Policy Checklist.

Statistics

For all statistical analyses, confirm that the following items are present in the figure legend, table legend, main text, or Methods section.

n/a	Confirmed	
☐	☒	The exact sample size (*n*) for each experimental group/condition, given as a discrete number and unit of measurement
☐	☒	A statement on whether measurements were taken from distinct samples or whether the same sample was measured repeatedly
☐	☒	The statistical test(s) used AND whether they are one- or two-sided<br>*Only common tests should be described solely by name; describe more complex techniques in the Methods section.*
☐	☒	A description of all covariates tested
☒	☐	A description of any assumptions or corrections, such as tests of normality and adjustment for multiple comparisons
☐	☒	A full description of the statistical parameters including central tendency (e.g. means) or other basic estimates (e.g. regression coefficient) AND variation (e.g. standard deviation) or associated estimates of uncertainty (e.g. confidence intervals)
☐	☒	For null hypothesis testing, the test statistic (e.g. *F*, *t*, *r*) with confidence intervals, effect sizes, degrees of freedom and *P* value noted<br>*Give P values as exact values whenever suitable.*
☒	☐	For Bayesian analysis, information on the choice of priors and Markov chain Monte Carlo settings
☒	☐	For hierarchical and complex designs, identification of the appropriate level for tests and full reporting of outcomes
☒	☐	Estimates of effect sizes (e.g. Cohen's *d*, Pearson's *r*), indicating how they were calculated

Our web collection on statistics for biologists contains articles on many of the points above.

Software and code

Policy information about availability of computer code

Data collection	No software was used for data collection.
Data analysis	For the integration of the Plant Lipid Module a set of custome codes were written in Matlab (R2022a). The codes are interfaced with the COBRA Toolbox v3.0, the set of Matlab functions 'Stoichiometry Tools', and the Gurobi Optimizer for Matlab (version 8.0.1). The analysis and processing of lipidomics data were done with the commercial software REFINER MS® 10.0 (GeneData, http://www.genedata.com) and XCalibur (Version 3.0, Thermo-Fisher, Bremen, Germany). For the simulation analyses, a set of custom codes were written in Matlab, which in some specific cases were interfaced with the COBRA Toolbox v3.0, and the Gurobi Optimizer. For the Genome-Wide Association Study the R package rMVP was used (R version 4.2.1.). The software for the integration of the PLM is available online in GitHub: https://github.com/marce2336/PlantLipidModule.git.

For manuscripts utilizing custom algorithms or software that are central to the research but not yet described in published literature, software must be made available to editors and reviewers. We strongly encourage code deposition in a community repository (e.g. GitHub). See the Nature Portfolio guidelines for submitting code & software for further information.

Data

Policy information about availability of data

All manuscripts must include a data availability statement. This statement should provide the following information, where applicable:

- Accession codes, unique identifiers, or web links for publicly available datasets
- A description of any restrictions on data availability
- For clinical datasets or third party data, please ensure that the statement adheres to our policy

For the reconstruction of the PLM the following databases were consulted: Aralipid website (http://aralip.plantbiology.msu.edu/), the Kyoto Encyclopedia of Genes and Genomes (KEGG) (https://www.kegg.jp/), the enzyme repository BRENDA (https://www.brenda-enzymes.org/), the universal protein database (UniProt) (https://www.uniprot.org/), the Arabidopsis Information Resource (TAIR) (https://www.arabidopsis.org/), the Arabidopsis Information Portal (https://www.araport.org) and the central resource for Arabidopsis protein subcellular location data (SUBA) (https://suba.live/). The data supporting the findings of this study are available in the article and its Supplementary Data files. The source data underlying Figures 2-5 are provided as the corresponding Source Data files.

Human research participants

Policy information about studies involving human research participants and Sex and Gender in Research.

Reporting on sex and gender	The present study did not involve research on humans.
Population characteristics	N/A
Recruitment	N/A
Ethics oversight	N/A

Note that full information on the approval of the study protocol must also be provided in the manuscript.

Field-specific reporting

Please select the one below that is the best fit for your research. If you are not sure, read the appropriate sections before making your selection.

☒ Life sciences ☐ Behavioural & social sciences ☐ Ecological, evolutionary & environmental sciences

For a reference copy of the document with all sections, see nature.com/documents/nr-reporting-summary-flat.pdf

Life sciences study design

All studies must disclose on these points even when the disclosure is negative.

Sample size	A population of 284 Arabidopsis natural accessions of the HapMap panel was used in this study for which metabolite profiles (e.g. sugars, organic acids, amino acids, and lipids) were measured. The use of the HapMap panel guarantees the maximization of diversity and to minimize redundancy and close family relatedness.
Data exclusions	The computational analyses were based on data measured in the 284 Arabidopsis accessions, and on publicly available data, and none of them were excluded from analyses.
Replication	The simulation findings were validated using experimental data available on public databases and peer reviewed publications.
Randomization	The Arabidopsis accessions were grown on independent 6-cm pots, that were placed randomly to avoid block effects during growth. Each tray contained one Col-0 plant to monitor the extent of spatial variation across trays. PCA was conducted for metabolites using all accessions, where Col-0 plants collected at the same time point clustered closely, indicating minor variation.
Blinding	The Arabidopsis accessions were identified with codes in such a way that both the sampling and the subsequent analyzes were carried out without the researchers having knowledge of the information associated with each accession.

Reporting for specific materials, systems and methods

We require information from authors about some types of materials, experimental systems and methods used in many studies. Here, indicate whether each material, system or method listed is relevant to your study. If you are not sure if a list item applies to your research, read the appropriate section before selecting a response.

Materials & experimental systems

n/a	Involved in the study
☒	☐ Antibodies
☒	☐ Eukaryotic cell lines
☒	☐ Palaeontology and archaeology
☒	☐ Animals and other organisms
☒	☐ Clinical data
☒	☐ Dual use research of concern

Methods

n/a	Involved in the study
☒	☐ ChIP-seq
☒	☐ Flow cytometry
☒	☐ MRI-based neuroimaging
